# Genes involved in sex pheromone biosynthesis of *Ephestia cautella,* an important food storage pest, are determined by transcriptome sequencing

**DOI:** 10.1186/s12864-015-1710-2

**Published:** 2015-07-18

**Authors:** Binu Antony, Alan Soffan, Jernej Jakše, Sulieman Alfaifi, Koko D. Sutanto, Saleh A. Aldosari, Abdulrahman S. Aldawood, Arnab Pain

**Affiliations:** Department of Plant Protection, King Saud University, Chair of Date Palm Research, College of Food and Agricultural Sciences, Riyadh, 11451 Saudi Arabia; Department of Plant Protection, King Saud University, EERU, Riyadh, Saudi Arabia; Agronomy Department, University of Ljubljana, Biotechnical Faculty, SI-1000 Ljubljana, Slovenia; BASE Division, KAUST, Thuwal, Jeddah, 23955-6900 Saudi Arabia

**Keywords:** *Ephestia*, Pheromone, Pheromone gland, Transcriptome, Pheromone biosynthetic enzymes

## Abstract

**Background:**

Insects use pheromones, chemical signals that underlie all animal behaviors, for communication and for attracting mates. Synthetic pheromones are widely used in pest control strategies because they are environmentally safe. The production of insect pheromones in transgenic plants, which could be more economical and effective in producing isomerically pure compounds, has recently been successfully demonstrated. This research requires information regarding the pheromone **biosynthetic pathways** and the characterization of pheromone biosynthetic enzymes (PBEs). We used Illumina sequencing to characterize the pheromone gland (PG) transcriptome of the Pyralid moth, *Ephestia cautella*, a destructive storage pest, to reveal putative candidate genes involved in pheromone biosynthesis, release, transport and degradation.

**Results:**

We isolated the *E. cautella* pheromone compound as (*Z,E*)-9,12-tetradecadienyl acetate, and the major pheromone precursors 16:acyl, 14:acyl, *E*14-16:acyl, *E*12-14:acyl and *Z*9,*E*12-14:acyl. Based on the abundance of precursors, two possible pheromone **biosynthetic pathways** are proposed. Both pathways initiate from C16:acyl-CoA, with one involving ∆14 and ∆9 desaturation to generate *Z*9,*E*12-14:acyl, and the other involving the chain shortening of C16:acyl-CoA to C14:acyl-CoA, followed by ∆12 and ∆9 desaturation to generate *Z*9,*E*12-14:acyl-CoA. Then, a final reduction and acetylation generates *Z*9,*E*12-14:OAc. Illumina sequencing yielded 83,792 transcripts, and we obtained a PG transcriptome of ~49.5 Mb. A total of 191 PBE transcripts, which included pheromone biosynthesis activating neuropeptides, fatty acid transport proteins, acetyl-CoA carboxylases, fatty acid synthases, desaturases, β-oxidation enzymes, fatty acyl-CoA reductases (FARs) and fatty acetyltransferases (FATs), were selected from the dataset. A comparison of the *E. cautella* transcriptome data with three other Lepidoptera PG datasets revealed that 45 % of the sequences were shared. Phylogenetic trees were constructed for desaturases, FARs and FATs, and transcripts that clustered with the ∆14, ∆12 and ∆9 desaturases, PG-specific FARs and potential candidate FATs, respectively, were identified. Transcripts encoding putative pheromone degrading enzymes, and candidate pheromone carrier and receptor proteins expressed in the *E. cautella* PG, were also identified.

**Conclusions:**

Our study provides important background information on the enzymes involved in pheromone biosynthesis. This information will be useful for the *in vitro* production of *E. cautella* sex pheromones and may provide potential targets for disrupting the pheromone-based communication system of *E. cautella* to prevent infestations.

**Electronic supplementary material:**

The online version of this article (doi:10.1186/s12864-015-1710-2) contains supplementary material, which is available to authorized users.

## Background

Pheromone-based methods of insect control are essential components of integrated pest management practices worldwide. The pheromones of over 2,000 insect species are now known, and The Pherobase is an updated compilation of pheromones and other behavior-modifying chemicals found in insects [1]. Common biosynthetic pathways have also been well-cited in many scientific publications over the last two decades, leading to production of species-specific pheromone compounds [2–5]. The female pheromones of almost all moth species are multicomponent blends of long hydrocarbon chains (10 to 18 carbons long), unbranched alcohols, and acetates or aldehydes, and are synthesized in the modified epidermal cells (pheromone-producing cells) from C16 or C18 fatty acid precursors [4, 6, 7]. A typical moth pheromone **biosynthetic pathway** begins even before the adult eclosion by releasing pheromone biosynthesis activating neuropeptide (PBAN) from the brain and transporting it to the pheromone gland (PG), which in turn activates functional group modification enzymes [3, 4, 8, 9] or acetyl-coenzyme A (CoA) carboxylase (ACC) [10]. As the first step in pheromone biosynthesis, carboxylation of acetyl-CoA to malonyl-CoA is catalyzed by ACC [10]. This is followed by fatty acid synthase (FAS) activity to produce saturated fatty acids (C18:0 and C16:0) using malonyl-CoA as the substrate. Later, the fatty acyl desaturases (DESs) introduce double bonds in the acyl chains, and then, specific β-oxidation enzymes shorten the chains. Once specific unsaturated pheromone precursors are formed, the terminal carboxyl group is modified to form one of the functional groups, alcohol, aldehyde or acetate ester (OH, CHO or OAc, respectively), and is catalyzed by fatty acyl reductase (FAR), aldehyde reductase (AR) or fatty acetyltransferase (FAT), respectively [3–5, 10]. A variety of desaturases, which introduce double bonds into the acyl at the ∆6 [11], ∆9 [12–14], ∆10 [15], ∆11 [13, 16, 17] and ∆14 [18] positions, have been cloned and functionally expressed from many moth species [2–5, 10]. Great progress has also been made in the functional characterization of FARs since their discovery in *Bombyx mori* [19] through detailed studies of pheromone evolution and the FARs of nine *Ostrinia* spp. [20–22], *Yponomeuta* spp. [23], *Helicoverpa* spp. and *Heliothis* spp. [24]. However, the molecular characterizations of other critical enzymes in the pheromone **biosynthetic pathway**, such as ACC, FAS, and several β-oxidation and acetylation enzymes, have not been characterized at the enzymatic level in insects.

Female moths typically start releasing sex pheromones a few days after emergence. In male moths, the pheromone molecule binds to odorant receptor (OR) proteins (in the antenna), signals are transmitted to the central nervous system where they are processed and identified by the brain, messages are then passed to the effector neurons, and finally the behavioral response is elicited. The expression of **OR** proteins is necessary and sufficient for odor detection in insects [25]. At first, volatile odors are bound to odorant-binding proteins (OBPs), a family that includes two sub-families, the pheromone-binding proteins (PBPs) and the general odorant-binding proteins (GOBPs) [26, 27]. Other important soluble secreted proteins that are found within the sensillum lymph include chemosensory proteins (CSPs) and the antennal binding protein X (ABPX) [28]. Finally, odorant molecules bind with **OR**s located in the dendritic membrane of receptor neurons [27, 29]. Sensory neuron membrane proteins (SNMPs) are another class of proteins involved in pheromone reception at the olfactory receptor neuron (ORN) [29–31]. Later, the signal termination is accomplished by the odorant-degrading enzymes (ODEs, also known as pheromone-degrading enzymes) [26, 32]. Knowledge of the olfactory communication system at the molecular level in insects is still in its early stages.

The tropical warehouse moth (almond moth), *Ephestia cautella* (Lepidoptera: Pyralidae) is a destructive polyphagous storage pest of wheat flour, dried figs, dates, nuts, chocolate, dried fruits, grain and associated processed food products worldwide. The control of these pests has depended exclusively on methyl bromide; however, methyl bromide was reported as facing an international phase-out by the year 2015 [33]. In this context, pheromones hold great potential in insect pest management [34]. In the last few decades, the elucidation of pheromone **biosynthetic pathways**, and the molecular characterization and functional gene expression of pheromone biosynthesis enzymes (PBEs) and OR proteins increased greatly [2, 10]. Most recently, through a synthetic biology approach, transgenic *Nicotiana benthamiana* plants with insect desaturases, FARs and FATs produced pure multi-component pheromone compounds [35]. Such *in vitro* production technology (green technology) could be cost effective and produce isomerically pure compounds that should be identical to chemically synthesized compounds. This research requires complete knowledge of the specific pheromone **biosynthetic pathway** and the functional characterization of enzymes (genes) involved in pheromone biosynthesis.

The rapid progress over the last decade resulted from the convergence of modern techniques from different areas of science has enriched our knowledge of the genetics of pheromone-based communications and olfactory communication systems. Transcriptome sequencing strategies are efficient for identifying a large number of expressed genes in specific tissues; thereby, providing information on the physiological, as well as molecular, properties of the tissue. Over the last few years, next-generation sequencing (NGS) techniques have provided easy and effective methods for the discovery of novel genes. These approaches are particularly relevant when no genomic data are available for the target species [36]. Over the past 5 years, RNA sequencing data on the insect pheromone gland amassed rapidly [37–41]. In the present study, using the Illumina sequencing approach, we constructed the transcriptome dataset of the PG of *E. cautella* and identified genes with putative roles in pheromone biosynthesis, transport and degradation. We combined the transcriptomic datasets with the *E. cautella* female sex pheromone precursors characterized through GC-MS studies, identified specifically or highly abundantly expressed genes in the PG and proposed roles for them in pheromone biosynthesis, binding, transport and release.

## Results and discussion

### Sex PG extraction and fatty-acyl precursor analysis

**Fig. 1:**
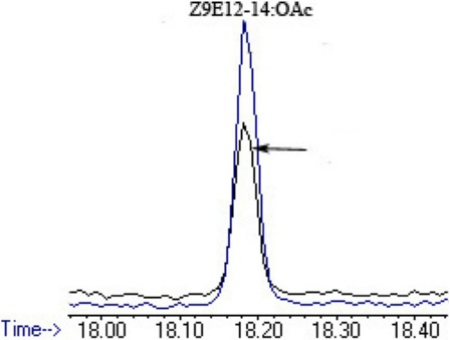
Pheromone compound analysis of PG extract from *E. cautella* by GC-MS

Analysis of the *E. cautella* PGs excised at the calling period (2-day-old, at mid-scotophase) showed the presence of the compound (*Z,E*)-9,12-tetradecadienyl acetate (*Z*9,*E*12-14:OAc) by their GC retention times (18.19 min) and mass spectra [ion fragment of *m/z* 61, a characteristic of acetate compounds (CH3COOH2+) and diagnostic ion at *m/z* 192] in comparison with those of authentic pheromone samples (Fig. 1). Our results were consistent with the earlier reports of the *E. cautella* female sex pheromone [34, 42]. Many studies have reported geographical variations and host-induced changes in the sex pheromone compounds and pheromone blend ratios in moths [2, 20, 21]. We isolated *E. cautella* (dried date fruit strain) sex pheromones to identify such differences. Date Palm (*Phoenix dactylifera* L) has been cultivated in Middle Eastern countries since ancient times, and *E. cautella* is native to Saudi Arabia where it infests dried date fruits in storage houses. To determine sex pheromone differences in the native moth strain, we studied its pheromone biosynthetic pathway as follows.

**Fig. 2:**
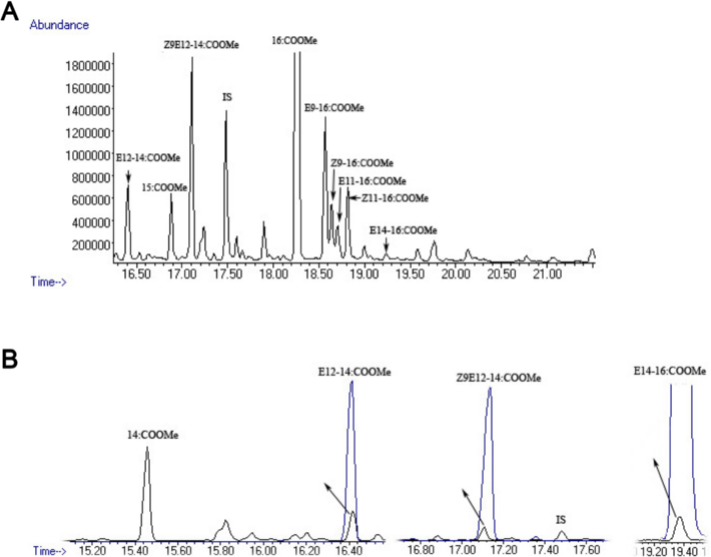
Fatty acid analysis of PG extracts from *E. cautella* by GC-MS. The fatty acids methyl ester (FAME) were identified based on the retention time (RT) of the authentic standard compound and mass spectra analysis

Fatty acid methyl esters (FAMEs) were made from the total lipid extract of *E. cautella* PG to determine the corresponding fatty acid precursors of *Z*9,*E*12-14:OAc. The PG extracts contained unsaturated and saturated FAMEs, such as methyl hexadecanoate (16:COOMe, related abbreviations used hereafter for similar FAMEs), 14:COOMe, 15:COOMe; *Z*9-16:COOMe; *E*9-16:COOMe; *Z*11-16:COOMe; *E*11-16:COOMe; 17:COOMe; 18:COOMe; *Z*9-18:COOMe and *Z*9,*Z*12-18:COOMe. The FAMEs were identified on the basis of their retention times relative to those of the authentic standards, as well as on their mass spectra [characterized by parent ions and an intense *m/z* = 74 and 87, M+, M+ −31, M+ −32, M+ −74 (example, C16: Me = 270, 239, 238, 196 and C14: Me = 242, 211, 210, 168, respectively)]. The GC-MS analysis of a methanolyzed gland lipid extracts showed the corresponding precursors, C16:acid, C14:acid, *E*14-16:acid, *E*12-14:acid and *Z*9,*E*12-14:acid (Fig. 2a). The mono- and di-unsaturated precursor GC retention times and mass spectra matched those of the authentic standard samples (Fig. 2b). When comparing the relative proportions of the derived acids, *E*12-14:acid appeared to be more abundant. There were also large amounts of other FAMEs identified as those of *E*9-16:COOMe and *Z*9-16:COOMe, as well as small amounts of others tentatively assigned as *E*11-16:COOMe and *Z*11-16:COOMe (Fig. 2a) (diagnostic ions at *m/z* 242 for 14:COOMe, *m/z* 240 for *E*12-14:COOMe, *m/z* 270 for 16:COOMe, *m/z* 268 for *Z*9-, *E*9-, *E*11- and *Z*11-16:COOMe, *m/z* 252 [M+ −32] and 284 [M+] for *E*14-16:COOMe and *m/z* 206 [M+ −32] and *m/z* 238 [M+] for *Z*9,*E*12-14:COOMe).

**Fig. 3:**
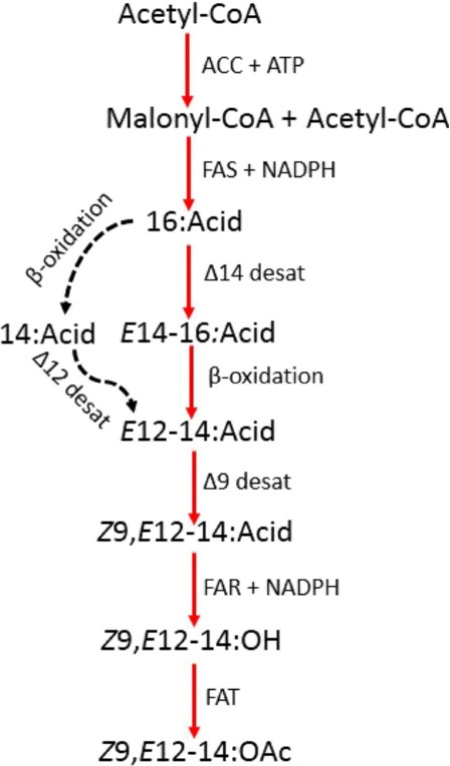
Proposed pheromone biosynthetic pathway leading to the sex pheromone of *E. cautella*, (*Z,E*)-9,12-tetradecadienyl acetate

Based on the identified pheromone precursors, the putative sex pheromone **biosynthetic pathway** of *E. cautella* was predicted as shown in Fig. 3. In *E. cautella* five major pheromone precursors, C14:acid, C16:acid, *E*14-16:acid; *E*12-14:acid and *Z*9,*E*12-14:acid, were identified using the FAME analysis of the PG. Thus, it is rational to propose a pheromone **biosynthetic pathway** in which the saturated fatty acid precursor of the *E. cautella* sex pheromones is palmitic acid (16:0) that is desaturated by Δ14-desaturase to form the pheromone precursor *E*14-16:acyl-CoA, which in turn has its chain shortened by β-oxidation to *E*12-14:acyl-CoA. A unique Δ9-desaturase uses the *E*12-14:acyl-CoA to produce *Z*9,*E*12-14:acyl-CoA that is reduced and acetylated to form *Z*9,*E*12-14:OAc, the final pheromone compound (Fig. 3). An alternative pathway can also be proposed that involves the chain shortening of C16:acyl-CoA to C14:acyl-CoA, which is later desaturated by Δ12-desaturase to produce *E*12-14:acyl-CoA, and then a unique Δ9-desaturase uses the *E*12-14:acyl-CoA to produce *Z*9,*E*12-14:acyl-CoA (Fig. 3). This is reduced and acetylated to form *Z*9,*E*12-14:OAc, the final pheromone compound of *E. cautella*. Based on the FAME analysis, the first pathway appears more fitting; however, further studies using *in vivo* labelling are required to test the hypothesis. We compared the *E. cautella* pheromone **biosynthetic pathway** with two *Spodoptera* spp., *S. exigua* and *S. littoralis*, that use *Z*9,*E*12-14:OAc as a sex pheromone compound (see Additional file 1: Figure S1). In the present study, we isolated the sex pheromone, *Z*9,*E*12-14:OAc from *E. cautella* infesting dried date fruit and identified a major pheromone precursor *E*12-14:acid. Hence, the proposed pheromone **biosynthetic pathway** (Fig. 3) appears to be more appropriate. The common **biosynthetic pathway** leading to the production of a moth sex pheromone compound, based on the activity of a desaturase with a strict regio- and stereo-selectivity, produced different pheromone precursors, which are characteristic of different species (Additional file 1: Figure S1) [3, 11–16].

### Illumina sequencing and *de novo* assembly

Illumina sequencing of a cDNA library prepared from mRNA of the *E. cautella* PG produced 237,048,152 raw reads with an average length of 101 base pairs (bp). After trimming adaptor sequences and eliminating low quality reads, there were 231,851,937 reads (227,994,544 sequences in pairs and 3,857,393 single sequences) with an average length of 100 bp (Additional file 2: Table S2). The raw reads were deposited in the National Center for Biotechnology Information (NCBI) Sequence Read Archive (SRA) database with the accession number SRX646348. After assembly, with scaffolding, 83,792 transcripts with an average length of 590 bp were obtained, having a maximum length of 19,518 bp. Most transcripts had lengths that ranged from 376 to 760 bp. The whole transcriptome size was 49.5 Mb, and the N50 size was 760 bp, with 10,856 sequences longer than 1 kb. This Transcriptome Shotgun Assembly project has been deposited at DDBJ/EMBL/GenBank under the accession GBXH00000000. In comparison with previously reported PG transcriptome/EST data [37–41], this pooled assembly of *E. cautella* PG sequences has the second greatest data volume and sequence lengths (Additional file 2: Table S2).

### Functional annotation

The assembled transcripts were used as query in a BLASTx against the non-redundant (*nr*) NCBI protein database, UniProtKB, Flybase and KEGG, all with an e-value cut-off of 10E − 5. Most of the sequences had an e-value between 1.0E − 4 and 1.0E − 10 (Additional file 3: Figure S3A). The similarity between *E. cautella* PG sequences and those of the databases ranged from 36 % to ~100 % (value: 3,434) with a peak at 65 % (value: 12,152) (Additional file 3: Figure S3B). A BLAST2GO analysis of the 83,792 transcripts of the *E. cautella* PG resulted in 30,582 transcripts with blast hits, 53,210 without blast hits, 4,217 with mapping results and 20,615 annotated sequences (Additional file 4: Figure **S**4A). The sequences without blast hits may have low similarities to functionally similar genes in the database, novel genes or parts of the 5′ or 3′ UTR regions. The PG transcript of *E. cautella* produced the most significant hits to *B. mori* sequences, followed by *Danaus plexippus* sequences (Additional file 3: Figure S3C). The evidence code distribution for the BLAST hit chart indicates an over-representation of Inferred Electronic Annotation (IEA), followed by Inferred by Mutant Phenotype (IMP) and Inferred by Direct Assays (IDAs) (Additional file 5: Figure S5A). The maximum evidence code for the individual sequences was through IEA, IMP and lastly IDA (Additional file 5: Figure S5B). The majority of functional predictions from the coding sequences were obtained from UniProtKB followed by FlyBase (FB) (1,017,318 and 107,608, respectively) (Additional file 5: Figure S5C).

GO terms were assigned by BLAST2GO through a search of the *nr* database, and INTERPRO was searched using INTERPROSCAN, resulting in ~34,953 transcripts from INTERPRO and 48,838 transcripts ‘without INTERPRO’ that had GO-annotation average lengths of 590 bp. Using this method, 12,455 unigenes were assigned to one or more GO terms. ANNEX was run after BLAST, and INTERPROSCAN results were annotated with the following results: 105,242 total original annotations, 7,810 new annotations, 1,007 original annotations replaced by new annotations due to specificity, and 3,853 confirmed annotations.

As shown in Table 1, 30,097 genes, 35 % of all transcripts in *nr*, 14,036 genes in UniProtKB, and 20,615 enzymes encoded in the Kyoto Encyclopedia of Genes and Genomes (KEGG) returned cut-off blast hits > 1.0E − 5. A KEGG metabolic pathway analysis revealed 5,762 transcripts could be assigned to generate 130 predicted pathways (Additional file 6). The major enzyme commission (EC) classes included oxidoreductases (964 transcripts), transferases (2,576 transcripts), hydrolases (2,125 transcripts), lyases (200 transcripts), isomerases (125 transcripts) and ligases (391 transcripts). The KEGG pathway map revealed the presence of a large number of PG transcripts involved in fatty acid biosynthesis (42 transcripts, 8 enzymes), fatty acid elongation (34 transcripts, 6 enzymes), fatty acid degradation (97 transcripts, 15 enzymes) and most importantly, the biosynthesis of unsaturated fatty acids (Additional file 7: Figure S6) and genes (enzymes) that may participate in pheromone biosynthesis (pathway: see Fig. 3). In total, 65 transcripts encoding five enzymes, DES, FARs, FATs, Acyl-CoA oxidases and dehydrogenases, were assigned functional annotations (Additional file 7: Figure S6).

**Table 1:** Annotation of a pooled assembly, representing the *E. cautella* PG transcriptome

Database	Number of transcripts
*nr*	30097
UniProtKB	14036
InterPro	34953
GO	12455
KEGG	5762

### GO for the genes expressed in the *E. cautella* PG

**Fig. 4:**
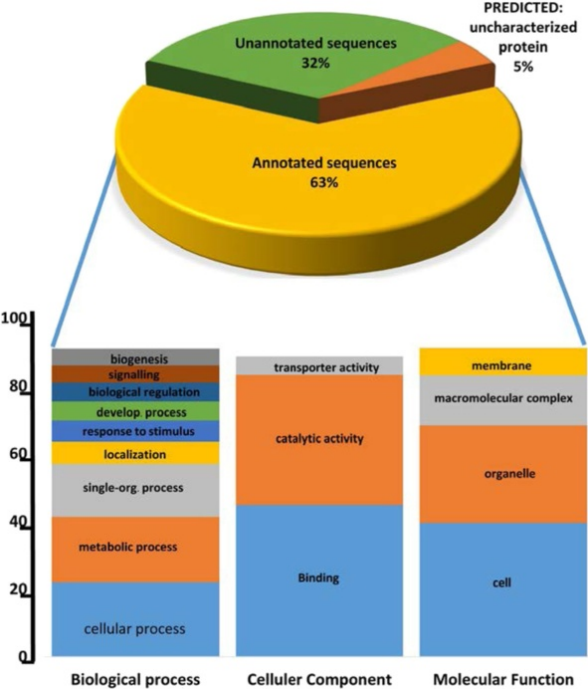
Pie and Stack chart showing the percentage of *E. cautella* predicted genes as annotated proteins, predicted proteins and unannotated proteins

**Fig. 5:**
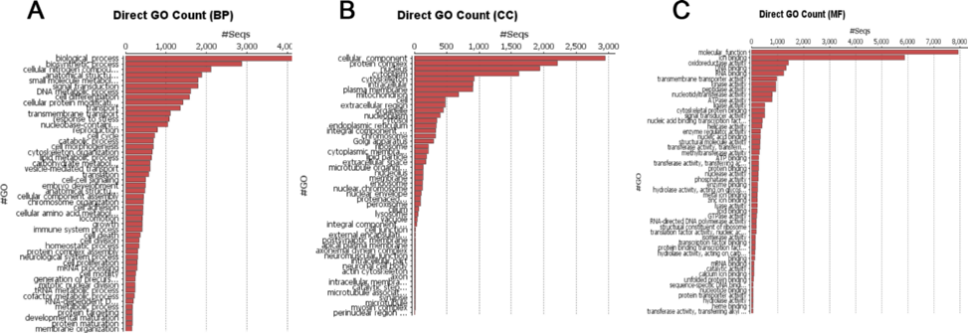
Distribution of enriched functions in **a**) Biological Process (BP), **b**) Molecular Functions (MF) and **c**) Cellular Component (CC)

Based on the matches to INTERPRO proteins, the *E. cautella* PG transcriptome was GO-annotated. The annotation results and distribution, GO-level distribution, number of GO-terms for *E. cautella* sequences with a specific length (*x*-axis), annotation score distribution and the percentage of *E. cautella* sequences with a specific length (*x*-axis), are depicted in Additional file 4: Figure S4. Among the total transcripts with BLAST results, 63 % were assigned GO terms, 32 % were unannotated proteins that had no matches in the GO database and 5 % were sequences assigned as predicted uncharacterized proteins (Fig. 4). The proteins with associated GO terms, such as “molecular function”, “biological process” and “cellular component” were grouped and recorded at different match levels (Fig. 4). The “cellular process” (10,559) and “metabolic process” (9,305) GO categories had the most abundant transcripts within the “biological process” GO ontology (Fig. 4). In the “cellular components” the most abundant transcripts were in “binding” (9,941) and “catalytic activity” (8,479) (Fig. 4). In the “cellular components” the transcripts were mainly distributed in “cell” (7,207) and “cell part” (7,123) (Fig. 5). In the “molecular function” ontology, 9,941 transcripts with “binding” functions were annotated, as were 8,479 that had “catalytic activity” (Fig. 5c). Of the proteins that had matches to the *nr* database, the most abundant protein class was the binding proteins. Other highly abundant proteins included oxidoreductase proteins, kinases, peptidases, cytoskeletal proteins, ribosomal proteins and proteins involved in other major functional categories (Fig. 5c). Of the direct GO counts identified for “biological process”, lipid metabolic process (including pheromone biosynthesis) and reproduction were among the first 20 dominant terms (Fig. 5a). Of the categories enriched for the direct GO counts identified as “cellular component”, the protein complex, nucleus and cytoplasm were the largest groups (Fig. 5b).

### Transcript abundance in the *E. cautella* PG

The highly expressed transcripts in the *E. cautella* PG are summarized in Table 2. The most abundant transcripts included vitellogenin and vitellogenin precursor (Total read count: 1,646,398 and 148,469, respectively), a major reproductive protein and its precursor, respectively, in insect egg production [43]. The results were consistent with a previous report of transcriptionally abundant proteins in *Agrotis ipsilon*’s PG [39]. The acyl-CoA desaturases, contigs 349 and 1,286, were highly expressed in the PG with 3,656 and 4,967 reads per kilobase per million reads (RPKM), respectively, indicating their roles in pheromone biosynthesis. Other highly abundant transcripts were of contigs 88 and 106 with 7,575 and 3,711 RPKMs, respectively, encoding CSPs that exhibited a 61 % identity with *Sesamia inferens* (Genbank: AGY49267) [40] and 62 % with *A. ipsilon* (Genbank: AGR39573) [39], respectively. The major housekeeping genes, such as elongation factor, cytochrome c oxidase subunit I and III, and circadian clock-controlled protein (period gene), were highly expressed in the PG of *E. cautella* (Table 2).

**Table 2:** The most abundant mRNAs in the *E. cautella* PG

Name	Accession no.	Sequence description	Species	Accession number	RPKM	*E*-value	% identity	Total read count
EP_contig_ 52	GBXH01000147	Vitellogenin	*Actias selene*	ABP63663	13916	3e-61	45.9	1646398
EP_contig_ 537	GBXH01000631	Vitellogenin	*Helicoverpa armigera*	AGL08685	13631	8e-32	56.41	798415
EP_contig_ 1252	GBXH01001346	Vitellogenin	*Actias selene*	ADB94560	13436	1e-27	60	722334
EP_contig_ 122	GBXH01000217	Vitellogenin	*Actias selene*	ADB94560	12643	1e-63	51.57	1401398
EP_contig_ 360	GBXH01000454	Vitellogenin	*Bombyx mandarina*	BAE47146	7888	6e-55	37.54	579822
EP_contig_ 88	GBXH01000183	Putative chemosensory protein	*Sesamia inferens*	AGY49267	7575	1e-39	61.16	476401
EP_contig_ 695	GBXH01000789	Vitellogenin	*Cnaphalocrocis medinalis*	AEM75020	7229	4e-70	72.11	531384
EP_contig_ 1286	GBXH01001379	Delta 11 desaturase	*Amyelois transitella*	AGO96562	4967	4e-68	6365	393132
EP_contig_ 73	GBXH01000168	Juvenile hormone binding protein precursor-like protein	*Manduca sexta*	AAF16700	4835	1e-81	53.36	793432
EP_contig_ 1468	GBXH01001560	Hypothetical protein KGM_06638	*Danaus plexippus*	EHJ78007	4821	1e-05	49.15	544825
EP_contig_ 114	GBXH01000209	BCP inhibitor precursor	*Bombyx mori*	NP_001037057	4705	3e-28	49.02	348204
EP_contig_ 100	GBXH01000195	Elongation factor 1-a	*Spodoptera litura*	AGC82213	3754	0.0	99.3	1031543
EP_contig_ 50	GBXH01000145	Cytochrome c oxidase subunit I, (mitochondrion)	*Ephestia kuehniella*	YP_008593341	3714	0.0	88.88	1604856
EP_contig_ 106	GBXH01000201	Chemosensory protein 3	*Agrotis ipsilon*	AGR39573	3711	3e-39	62.39	223555
EP_contig_ 349	GBXH01000443	Delta 11 desaturase	*Amyelois transitella*	AGO96562	3656	3e-80	79.87	1098774
EP_contig_ 2843	GBXH01002931	Putative chemosensory protein	*Sesamia inferens*	AGY49266	3592	8e-06	53.8	217586
EP_contig_ 306	GBXH01000400	Circadian clock-controlled protein-	*Bombyx mori*	XP_004932669	3245	9e-33	65.56	553500
EP_contig_ 243	GBXH01000337	Cytochrome c oxidase subunit III	*Ephestia kuehniella*	YP_008593345	3192	4e-112	81.78	863353
EP_contig_ 967	GBXH01001061	Vitellogenin precursor	*Bombyx mori*	NP_001037309	2687	3e-49	72.4	148469

### Comparative analysis of PG transcripts in Lepidoptera

By comparing *E. cautella* PG transcripts with those of *B. mori* and *H. virescens* from the NCBI database of differentially expressed transcripts and *A. ipsilon* from the SRA database, a large number of PG transcriptome sequences were found to be homologous. After assembly, we obtained 17,508 unigenes from *A. ipsilon* and 11,001 and 13,612 ESTs from *B. mori* and *H. virescens*, respectively.

**Fig. 6:**
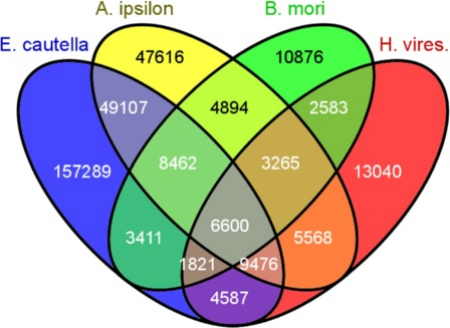
Venn diagram showing the comparative analysis of the *E. cautella* PG transcriptome with those of *A. ipsilon, B. mori* and *H. virescens*

We selected the first 10 bidirectional hits for each transcript from *E. cautella*, *A. ipsilon*, *H. virescens* and *B. mori* (producing a total of 240,753, 134,988, 46,912 and 46,940 blast hit results, respectively) for the comparative analysis. When comparing the PG transcripts pairwise using the bidirectional blast hit results, we found that between *E. cautella* and the three other Lepidoptera, 45 % of the blast hits were shared, and 65 % of the blast hits were unique to *E. cautella* (Fig. 6). The comparison between *E. cautella* and *A. ipsilon* showed that 20 % of the blast hits were shared, and 65 % of the blast hits were unique to *E. cautella* (Fig. 6). Similarly, a comparative analysis of blast hits of *E. cautella*, *A. ipsilon* and *B. mori* showed that 3.5 % of the blast results were shared. Comparative blast hits of *E. cautella*, *A. ipsilon* and *H. virescens* showed that 3.9 % had homologous hits, while between *E. cautella* and *H. virescens* there was 1.9 %, and between *E. cautella* and *B. mori* there was 1.4 % shared blast hits (Fig. 6). A large portion of the *E. cautella* transcripts (65 %) had no homologous hits in the available PG transcriptomes/ESTs of the other three species. This may have been because of the larger data set (83,792 transcripts) for *E. cautella* and the lower coverage in the other studies (Fig. 6). The high number of *E. cautella* blast hit results, which did not match *A. ipsilon*, *B. mori* or *H. virescens* may be due to novel genes with unique functions or highly conserved genes.

### Identification of candidate genes involved in pheromone biosynthesis

In the present study, the *E. cautella* pheromone compound identified was *Z*9,*E*12-14:OAc, and the pheromone **biosynthetic pathway** is likely to be similar to those in other Pyralid moths (or type I pheromone biosynthesis), which include fatty acid synthesis (ATP-dependent carboxylation and decarboxylation condensation with several malonyl moieties), including the actions of desaturases and β-oxidation enzymes, followed by modifications of the carboxyl group by reductases and acetyltransferases [44]. Using BLASTx searches, we identified members of gene subfamilies in the *E. cautella* PG transcriptome putatively involved in *Z*9,*E*12-14:OAc pheromone production (Table 3). These include two PBAN receptor isoforms, five fatty acid transport proteins (FATPs), six ACCs, 12 FASs, 22 DESs, 28 FARs, 18 FATs and 11 ARs (Table 3). Additionally, 87 transcripts encoding putative β*-*oxidation enzymes, including 28 acyl-CoA dehydrogenases, 17 acyl-CoA oxidases, 13 enoyl-CoA hydratases, 17 L-3-hydroxyacyl-CoA dehydrogenases, eight 3-ketoacyl-CoA thiolases, three delta-3, delta-2 trans-enoyl-CoA isomerases and a delta(3,5)-delta(2,4)-dienoyl-CoA isomerase, were identified (Additional file 8: Table S7). There were also 36 transcripts encoding putative pheromone degrading enzymes (Additional file 9: Table S8), three transcripts encoding putative ABPs, 17 transcripts encoding putative OBPs, seven candidate CSPs, two transcripts encoding PBPs, 21 candidate **OR**s, two candidate sensory neuron membrane proteins and three candidate ionotropic receptors (IRs) (Additional file 10: Table S9 and Additional file 11: Table S10). Their abundance levels, based on RPKM values, in the PG transcriptome are shown in Table 3.

**Table 3:** Putative pheromone biosynthesis enzymes (PBEs) in the *E. cautella* PG

Unigene	Accession no.	Length (bp)	Putative identification	Species	Accession no.	Blast Hit score	E-value	% of identity	RPKM
PBAN receptor									
EP_Contig_27375_PBAN	GBXH01027379	3094	PBAN receptor isoform C	*Ostrinia nubilalis*	AGL12068	479	1.00E-155	63.1	4.39
EP_Contig_24961_PBAN	GBXH01024977	482	PBAN receptor isoform A	*Ostrinia nubilalis*	AGL12066	103	4.00E-23	60.1	0.86
Fatty acid Transport Protein									
EP_Unigene_1_FATP	GBXH01082863	2057	long chain fatty acid transport protein 1	*Bombyx mori*	XP_004927673	675	0	67.6	46.52
EP_Unigene_2_FATP	GBXH01082864	1917	long chain fatty acid transport protein 4	*Nasonia vitripennis*	XP_001603871	668	0	73.8	100
EP_Unigene_3_FATP	GBXH01082865	966	Fatty acid transport protein	*Ostrinia scapulalis*	BAJ33524	555	0	80.8	140
EP_Contig_1647_FATP	GBXH01001738	366	Fatty acid transport protein	*Eilema japonica*	BAJ33523	190	3.78E-54	79.9	120
EP_Contig_12202_FATP	GBXH01012261	526	Fatty acid transport protein	*Papilio xuthus*	BAM19873	157	5.88E-44	81.4	33
Acetyl CoA carboxylase									
EP_Unigene_1_ACC	GBXH01000029	1938	Acetyl CoA carboxylase isoform b	*Agrotis ipsilon*	AGR49308	926	0	83.4	122
EP_Unigene_2_ACC	GBXH01000030	1464	Acetyl CoA carboxylase	*Agrotis ipsilon*	AGR49308	834	0	84.8	91
EP_Unigene_3_ACC	GBXH01000031	1342	Acetyl CoA carboxylase-like	*Bombyx mori*	XP_004930758	803	0	93.4	70
EP_Unigene_4_ACC	GBXH01000032	607	Acetyl CoA carboxylase	*Danaus plexippus*	EHJ73343	394	1.40E-126	89.8	64
EP_Contig_63068_ACC	GBXH01062648	205	Acetyl CoA carboxylase	*Agrotis ipsilon*	AGR49309	64	5.50E-11	77.5	9
EP_Contig_14940_ACC	GBXH01014992	776	Acetyl CoA carboxylase	*Agrotis ipsilon*	AGR49308	300	8.70E-89	67.4	74
Fatty acid synthase									
EP_Contig_284_FAS	GBXH01000378	3493	fatty acid synthase	*Agrotis ipsilon*	AGR49310	1254	0	67.5	53
EP_Contig_1101_FAS	GBXH01001195	2074	putative fatty acid synthase	*Danaus plexippus*	EHJ78836	473	5.22E-143	68.6	109
EP_Contig_8286_FAS	GBXH01008363	1991	fatty acid synthase	*Agrotis ipsilon*	AGR49310	1127	0	82.3	51
EP_Contig_42681_FAS	GBXH01042586	718	fatty acid synthase-like	*Bombyx mori*	XP_004927661	73	2.21E-11	51	0.3
EP_Contig_55530_FAS	GBXH01055262	1079	fatty acid synthase-like	*Bombyx mori*	XP_004922805	228	6.32E-61	55.2	0.7
EP_Contig_69718_FAS	GBXH01069102	665	fatty acid synthase-like	*Bombyx mori*	XP_004925618	137	9.52E-33	55.8	0.39
EP_Contig_72627_FAS	GBXH01071942	325	fatty acid synthase-like	*Bombyx mori*	XP_004922805	105	1.68E-23	65.3	0.12
EP_Contig_74719_FAS	GBXH01073973	714	fatty acid synthase-like	*Bombyx mori*	XP_004925618	179	2.99E-47	54.7	0.33
EP_Contig_74831_FAS	GBXH01074081	260	fatty acid synthase-like	*Bombyx mori*	XP_004925618	121	2.49E-26	68.2	0.18
EP_Contig_76686_FAS	GBXH01075900	518	fatty acid synthase-like	*Bombyx mori*	XP_004925618	146	1.19E-36	59.4	0.18
EP_Contig_79616_FAS	GBXH01078764	345	fatty acid synthase-like	*Bombyx mori*	XP_004922805	72	6.27E-12	59.1	0.12
EP_Contig_81802_FAS	GBXH01080886	342	fatty acid synthase-like	*Bombyx mori*	XP_004922805	170	4.53E-46	83.6	0.15
Desaturase									
EP_Unigene_3_DES	GBXH01000080	868	desaturase-like protein oblr-fb7a	*Choristoneura rosaceana*	AAN39698	130	5.38E-60	88.2	284
EP_Unigene_4_DES	GBXH01000081	744	terminal desaturase	*Amyelois transitella*	AGO96562	452	2.30E-157	86.1	3391
EP_Unigene_7_DES	GBXH01000082	1227	desaturase-like protein sfwg-nf-b	*Ctenopseustis herana*	AER29846	376	1.04E-124	68.8	261
EP_Unigene_9-1286_DES	GBXH01000083	932	terminal desaturase	*Amyelois transitella*	AGO96562	221	1.14E-135	79.3	4967
EP_Unigene-10_DES	GBXH01000084	875	stearoyl-coa desaturase	*Bombyx mori*	NP_001274329	357	4.81E-119	66.4	38
EP_Unigene11_14851DES	GBXH01000085	678	acyl- z9 desaturase	*Agrotis ipsilon*	AGR49313	150	1.64E-65	81	55
EP_Unigene_12_DES	GBXH01000086	636	acyl- delta desaturase	*Bombyx mori*	XP_004932163	333	7.73E-112	82	0.6
EP_Contig_ 343_DES	GBXH01000437	2168	delta 11 desaturase	*Amyelois transitella*	AGO96562	288	4.00E-87	70.56	1739
EP_Contig_ 5930_DES	GBXH01006012	1002	Acyl-desaturase	*Bombyx mori*	XP_004929766	364	7.73E-121	86.7	41
EP_Contig_ 20984_DES	GBXH01021017	874	acyl-delta-9 desaturase	*Manduca sexta*	CAJ27975	363	3.07E-134	95.6	1.82
EP_Contig_ 25772_DES	GBXH01025783	405	acyl-delta-9-3a-desaturase	*Danaus plexippus*	EHJ76461	150	6.39E-41	89	19
EP_Contig_ 63178_DES	GBXH01062755	207	Acyl-desaturase	*Heliothis virescens*	AGO45840	131	1.50E-36	96.3	0.98
EP_Contig_ 69106_DES	GBXH01068508	631	acyl-delta desaturase-like	*Bombyx mori*	XP_004925564	160	2.74E-43	68.5	0.74
EP_Contig_ 81260_DES	GBXH01080359	330	acyl-z6 desturase	*Lampronia capitella*	ABX71630	79	2.96E-15	58.9	0.16
EP_Contig_ 37061_DES	GBXH01036958	1551	acyl-delta desaturase-like	*Bombyx mori*	XP_004925564	206	4.96E-57	76.4	1.02
EP_Contig_ 27034_DES	GBXH01027039	1923	Acyl-desaturase	*Spodoptera littoralis*	AAQ74260	542	0	83.2	20
EP_Contig_ 36616_DES	GBXH01036561	379	acyl-delta desaturase-like	*Bombyx mori*	NP_001274329	57	3.07E-07	76	15
EP_Contig_ 70932_DES	GBXH01070286	612	desaturase	*Ostrinia nubilalis*	ADB25212	163	6.68E-45	70.7	0.32
EP_Contig_ 37918_DES	GBXH01037854	1028	Acyl-desaturase	*Spodoptera exigua*	AFO38465	399	5.46E-134	89	3
EP_Contig_ 71065_DES	GBXH01070415	724	acyl-detla9-4-desaturase	*Dendrolimus punctatus*	ABX71813	153	2.12E-40	70.4	0.2
EP_Contig_145_DES	GBXH01000239	330	terminal desaturase	*Ctenopseustis obliquana*	AER29852	267	2.00E-54	72.4	1752
Fatty Acyl Reductase									
EP_Unigene_ 1_FAR	GBXH01082835	1235	fatty-acyl CoA reductase 5	*Ostrinia nubilalis*	ADI82778	399	8.28E-131	77.3	743
EP_Unigene_ 2_FAR	GBXH01082836	1791	putative fatty acyl-CoA reductase CG8306-like isoform X1	*Bombyx mori*	XP_004930778	848	0	77	503
EP_Unigene_ 3_FAR	GBXH01082837	767	putative fatty acyl-CoA reductase CG5065-like	*Bombyx mori*	XP_004926012	117	5.08E-38	62	487
EP_Unigene_ 4_FAR	GBXH01082838	1529	fatty-acyl CoA reductase 1	*Ostrinia nubilalis*	ADI82774	581	0	77.1	41
EP_Unigene_ 5_FAR	GBXH01082839	1053	fatty-acyl CoA reductase 4	*Ostrinia nubilalis*	ADI82777	372	7.69E-122	73.1	74
EP_Unigene_ 6_FAR	GBXH01082840	933	fatty-acyl CoA reductase 2	*Ostrinia nubilalis*	ADI82775	466	3.48E-157	70.2	84
EP_Unigene_ 7_FAR	GBXH01082841	778	putative fatty acyl-CoA reductase CG5065-like	*Bombyx mori*	XP_004930776	385	5.10E-128	87.9	24
EP_Unigene_ 8_FAR	GBXH01082842	2493	putative fatty acyl-CoA reductase CG5065-like	*Bombyx mori*	XP_004930522	861	0	80.6	82
EP_Unigene_ 9_FAR	GBXH01082843	2019	fatty-acyl CoA reductase 5	*Danaus plexippus*	EHJ72233	223	8.62E-69	67.6	772
EP_Unigene_ 10_FAR	GBXH01082844	1280	putative fatty acyl-CoA reductase CG5065-like	*Bombyx mori*	XP_004925992	736	0	79.7	21
EP_Unigene_ 11_FAR	GBXH01082845	1203	fatty-acyl CoA reductase 6	*Ostrinia nubilalis*	ADI82779	492	5.84E-168	64.2	30
EP_Unigene_ 12_FAR	GBXH01082846	839	fatty-acyl CoA reductase 4	*Ostrinia nubilalis*	ADI82777	345	2.17E-112	73.4	65
EP_Unigene_ 14_FAR	GBXH01082847	594	putative fatty acyl-CoA reductase CG5065-like	*Danaus plexippus*	XP_004926010	108	1.47E-24	59.1	536
EP_Unigene_ 15_FAR	GBXH01082848	565	fatty-acyl CoA reductase 6, partial	*Agrotis ipsilon*	AGR49321	142	1.12E-37	65	3
EP_Contig_ 2421_FAR	GBXH01002511	433	Fatty-acyl CoA reductase 2	*Ostrinia nubilalis*	ADI82775	266	1.09E-83	86.71	41
EP_Contig_ 6194_FAR	GBXH01006275	1721	putative fatty acyl-CoA reductase CG5065-like	*Bombyx mori*	XP_004926017	516	5.33E-174	82.65	25
EP_Contig_ 45618_FAR	GBXH01045493	236	putative fatty acyl-CoA reductase CG5065-like	*Bombyx mori*	XP_004929542	58	5.32E-08	41.5	1
EP_Contig_ 11410_FAR	GBXH01011473	251	fatty-acyl CoA reductase 2	*Ostrinia nubilalis*	ADI82775	117	1.63E-28	67.5	30
EP_Contig_ 13590_FAR	GBXH01013646	516	putative fatty acyl-CoA reductase CG5065-like	*Bombyx mori*	XP_004929542	48	8.71E-09	50	43
EP_Contig_ 65474_FAR	GBXH01064989	483	putative fatty acyl-CoA reductase CG5065-like	*Bombyx mori*	XP_004925993	165	5.33E-45	75.69	0.5
EP_Contig_ 56254_FAR	GBXH01055966	473	fatty-acyl CoA reductase 5	*Danaus plexippus*	EHJ72233	177	3.18E-49	52.78	2
EP_Contig_ 10215_FAR	GBXH01010281	3493	putative fatty acyl-CoA reductase CG5065-like	*Bombyx mori*	XP_004930776	549	1.90E-179	81.94	15
EP_Contig_ 53541_FAR	GBXH01053315	1771	fatty-acyl CoA reductase 4	*Ostrinia nubilalis*	ADI82777	528	3.49E-179	53.76	0.6
EP_Contig_ 53189_FAR	GBXH01052971	1006	fatty-acyl CoA reductase 5	*Danaus plexippus*	EHJ72233	422	5.00E-141	63.21	2
EP_Contig_ 61889_FAR	GBXH01061492	326	putative fatty acyl-CoA reductase CG5065-like	*Bombyx mori*	XP_004929961	146	2.44E-39	79.49	0.24
EP_Contig_ 72742_FAR	GBXH01072052	225	putative fatty acyl-CoA reductase CG5065-like	*Bombyx mori*	XP_004929542	64	9.35E-10	40	0,16
EP_Contig_ 78653_FAR	GBXH01077818	271	FAR-like protein VI	*Ostrinia scapulalis*	ACJ06513	156	5.00E-32	58	0.3
EP_Contig_ 79681_FAR	GBXH01078826	384	putative fatty acyl-CoA reductase CG5065-like	*Bombyx mori*	XP_004925993	205	1.55E-60	81.89	0.25
Fatty acetyltransferase									
EP_Unigene_ 2_FAT	GBXH01082849	1054	Acetyltransferase 1 [cl21486]	*Danaus plexippus*	EHJ65205	371	7.20E-123	83.7	65
EP_Unigene_ 3_FAT	GBXH01082850	949	n-acetyltransferase esco1 [cl16450]	*Bombyx mori*	XP_004925351	366	1.79E-115	65	5
EP_Unigene_ 4_FAT	GBXH01082851	2303	n-alpha acetyltransferase [cl09317]	*Bombyx mori*	XP_004932434	648	0	78.1	28
EP_Unigene_ 5_FAT	GBXH01082852	1351	Acetyltransferase 1 [cl09938]	*Danaus plexippus*	EHJ65205	296	4.25E-92	65.3	2
EP_Unigene_ 6_FAT	GBXH01082853	916	Putative acetyltrasnferase [predicted]	*Danaus plexippus*	EHJ75659	124	4.28E-30	52.1	1.2
EP_Unigene_ 7_FAT	GBXH01082854	2115	n-acetyltrasnferase [cl17182]	*Danaus plexippus*	EHJ73917	350	8.18E-113	93.7	45
EP_Unigene_ 8_FAT	GBXH01082855	1577	n-acetyltrasnferase mak-3 like protein [cl17182]	*Bombyx mori*	XP_004928263	360	1.27E-117	78	43
EP_Unigene_ 9_FAT	GBXH01082856	1527	arylalkylamine n-acetyltrasnferase [cl17182]	*Biston betularia*	ADF43200	376	3.24E-125	73.9	91
EP_Unigene_ 10_FAT	GBXH01082857	1369	n-alpha-acetyltrasnferase 60-like [cl17182]	*Bombyx mori*	XP_004931652	471	1.90E-162	77.6	10
EP_Unigene_ 12_FAT	GBXH01082858	496	acetyltrasnfease1 [cl09938]	*Ostrinia scapulalis*	BAH03386	205	8.39E-61	90.2	112
EP_Unigene_ 13_FAT	GBXH01082859	495	n-alpha-acetyltransferase [predicted]	*Bombyx mori*	XP_004925677	188	1.07E-57	93.7	24
EP_Unigene_ 14_FAT	GBXH01082860	417	n-acetyltrasnferase 9-like protein [cl17182]	*Bombyx mori*	XP_004922983	155	1.72E-44	90.2	10
EP_Unigene_ 15_FAT	GBXH01082861	261	n-acetyltrasnferase 2 [predicted]	*Bombyx mori*	NP_001177771	152	5.12E-44	66.9	0.2
EP_Unigene_ 16_FAT	GBXH01082862	214	Acetyltrasnfease 1 [cl09938]	*Agrotis ipsilon*	AGQ45622	119	6.17E-31	85.7	9
EP_Contig_4335_FAT	GBXH01004419	496	acetyltransferase 1 [cl09938]	*Ostrinia scapulalis*	BAH03386	204	8.75E-61	92	113
EP_Contig_ 7673_FAT	GBXH01007751	927	acetyltrasnferase [cl17182]	*Agrotis ipsilon*	AGQ45625	360	7.31E-122	95	80
EP_Contig_ 18689_FAT	GBXH01018731	2885	n-alpha acetyltransferase [predicted]	*Bombyx mori*	XP_004922640	1403	0	83.1	3
EP_Contig_ 45366_FAT	GBXH01045245	540	n-acetyltransferase-40like [predicted]	*Bombyx mori*	XP_004921847	231	5.66E-73	77.55	8
Aldehyde reductase									
EP_Unigene_ 1_AR	GBXH01000069	460	Aldo-keto reductase	*Agrotis ipsilon*	XP_004925119	223	4.10E-69	82.2	30
EP_Unigene_ 2_AR	GBXH01000070	1177	Aldo-keto reductase	*Danaus plexippus*	EHJ72113	496	9.38E-172	72.5	27
EP_Unigene_ 4_AR	GBXH01000072	846	Aldo-keto reductase	*Agrotis ipsilon*	AGQ45621	209	9.97E-62	78.3	29
EP_Unigene_ 5_AR	GBXH01000073	1200	Aldo-keto reductase	*Chilo suppressalis*	AEW46852	487	6.43E-169	73.1	62
EP_Unigene_ 8_AR	GBXH01000074	577	Aldo-keto reductase	*Chilo suppressalis*	AEW46854	221	9.10E-68	71.5	22
EP_Unigene_ 9_AR	GBXH01000075	470	Aldo-keto reductase	*Papilio xanthus*	BAM20078	227	1.14E-70	75.7	84
EP_Contig_ 10669_AR	GBXH01010734	263	Aldo-keto reductase	*Bombyx mori*	XP_004926772	103	2.14E-24	68.9	2
EP_Contig_ 15787_AR	GBXH01015838	1025	Aldo-keto reducase 1	*Bombyx mori*	XP_004933321	415	6.30E-141	80.6	21
EP_Contig_ 19588_AR	GBXH01019627	244	Aldo-keto reductase	*Papilio xuthus*	BAM18493	117	2.12E-29	78.6	16
EP_Contig_ 62067_AR	GBXH01061668	400	Aldo-keto reductase	*Bombyx mori*	XP_004922743	211	3.99E-65	78	0.13
EP_Contig_ 39413_AR	GBXH01039339	205	Aldo-keto reducase domain containing protein	*Bombyx mori*	XP_004929974	107	7.13E-22	74.3	8

### The PBAN receptor

Previous studies concluded that the sex pheromone biosynthetic machinery of Lepidopteran PG cells is regulated by PBAN, which is released from the brain, goes to the hemolymph and binds to the PBAN receptor in the membrane of pheromone producing cells, triggering pheromone production [8, 9]. We found two transcripts, EP_contig_27375 and EP_contig_24961, encoding proteins highly homologous to PBAN receptor isoforms C and A, respectively (Table 3). They have very low abundance levels in the *E. cautella* transcriptome (4.38 and 0.8 RPKM) (Table 3) but high identities (60–63 %) to the *O. nubilalis* PBAN receptors C and A in GenBank (AGL12068 and AGL12066, respectively) [45]. The PBAN receptors functionally characterized from *O. nubilalis* [45] and *H. virescens* [46] include isoforms A and C, and in the present study we identified PBAN isoforms A and C from *E. cautella* PG, which should be involved in pheromone production. The sequence identity (93 %) of *E. cautella* PBAN isoforms A and C indicate that they are likely produced by alternative splicing at the 3′-end of the receptor gene as reported in other moths, generating multiple receptor subtypes [46]. We also found a G-protein-coupled receptor (EP_contig_34693) that shows homology (63 %) to the diapause hormone receptors of *H. zea* and *B. mori* (AGR34305 and NP_001036913, respectively). The diapause hormone receptor is a G-protein gamma-subunit homolog, which is hypothesized to interact with the PBAN receptor, and has been reported in the PG transcriptomes of *A. segatum* [37] and *H. virescens* [38].

### Fatty Acid Transport Protein (FATP) [EC:6.2.1.-]

FATPs belong to an evolutionarily conserved family of membrane-bound proteins that facilitate the uptake of extracellular long-chain fatty acids (LCFAs), and/or very LCFAs, and catalyze the ATP-dependent esterification of these fatty acids to their corresponding acyl-CoA derivatives [47]. The important role of FATPs in pheromonogenesis has been demonstrated in *B. mori* [47] and in *O. scapulalis* [48]. In *E. cautella,* we found five FATP isoforms in Unigene_3 (RPKM 140) with high transcript abundance levels and a high identity (80.8 %) to those of *O. scapulalis* (GenBank: BAJ33524) (Table 3).

### Acetyl CoA Carboxylase (ACC) [EC:6.4.1.-]

Pheromone biosynthesis begins with an ACC catalyzing the production of malonyl-CoA from acetyl-CoA in the first committed biosynthesis step [49, 50]. In the *E. cautella* PG we found six transcripts encoding ACCs. ACC partial sequence EP_unigene_1, 2 and EP_contig_14940 showed more than 90 % identity with *A. ipsilon* ACC (GenBank: AGR49308). EP_unigene_3_ACC showed 93 % similarity with *B. mori* ACC (GenBank XP_004930758) (Table 3). Based on their RPKM values (286), EP_unigene_1, 2 and EP_contig_14940 were relatively highly expressed in the *E. cautella* PG (Table 3).

### Fatty Acid Synthase (FAS) [EC:2.3.1.-]

In moth pheromone biosynthesis, FAS is supposed to catalyze the conversion of malonyl-CoA and NADPH to produce saturated fatty acids (16:acyl in *E. cautella*) [49]. We found 12 FAS-like partial transcripts in the *E. cautella* PG, which produced five different BLASTx hits in NCBI. Thus, we are proposing the existence of five FAS-like genes in *E. cautella* (Table 3). Partial sequences of EP_contig_284 and 8286 showed high similarity levels (<80 %) to *A. ipsilon* FAS (GenBank: AGR49310), whereas EP_contig_1101 showed a high similarity to *D. plexippus* FAS (GenBank: EHJ78836). The details of other FAS transcripts and BLASTx hit similarities are given in Table 3. Based on the RPKM value (110), EP_contig_1101 was highly expressed in the *E. cautella* PG (Table 3).

### Desaturases (DES) [EC:1.14.19.-]

The desaturases introduce a double bond into the fatty acyl carbon chain, with strict regio- and stereo-selectivity. The desaturases characterized thus far include enzymes that act on saturated and monounsaturated substrates, which include Δ5 [51], ∆6 [11], Δ9 [12–14, 52, 53], Δ10 [15, 54], Δ11 [13, 16, 17, 55, 56] and Δ14 [18, 57]. Desaturases are characterized by having three histidine boxes containing eight histidine residues, which are used for binding essential metal complexes used in the enzyme reaction, and acyl-CoA desaturases introduce unsaturated bonds into fatty acids that are bound to CoA [58].

In *E. cautella*, four major pheromone precursors, C14:acid; *E*14-16:acid; *E*12-14:acid and *Z*9,*E*12-14:acid, were identified using a FAME analysis of the PG. In the *E. cautella* sex pheromones’ biosynthesis, a two-step desaturation process is proposed, involving ∆14, or ∆12, and ∆9 desaturases (Fig. 3). In the *E. cautella* PG transcriptome, 22 transcripts encoding desaturases have been identified (Table 3). EP_unigene_9 and EP_contig_349 are the highly expressed desaturases in the *E. cautella* PG (4,967 and 3,657 RPKMs, respectively), followed by EP_unigene_4, and EP_contigs_145 and 343 (3,391, 1,752 and 1,739 RPKM, respectively) (Table 3). Contigs_349 and 45 are closely related to the *Ctenopseustis obliquana* desaturase (GenBank: AER29852), which has ∆9, ∆11 and ∆14 fatty acid desaturase activities [12]. EP_unigene_9_1286 and EP_contig_343 showed high similarities to *Amyelois transitella* (GenBank: AGO96562) and *O. furnacalis* Z/E11 desaturases (GenBank: AAL32060), respectively, which have ∆11 and ∆9 desaturase activities [15]. EP_contig_349 and Contig_145 may have ∆14 and ∆9 desaturase activities (multifunctional) and could be involved in the formation of *E*14-16:acyl-CoA and *Z*9,*E*12-14:acyl-CoA, and EP_unigene_9_1286, or EP_contig_343, may have ∆9 desaturase activity and could be involved in *Z*9,*E*12-14:acyl-CoA synthesis from *E*12-14:acyl-CoA or multifunctional ∆12 and ∆9 desaturase activities (Fig. 3). Further studies on the functional gene expression levels of these desaturases in a transformed yeast (*Saccharomyces cerevisiae*) expression system are in progress.

**Fig. 7:**
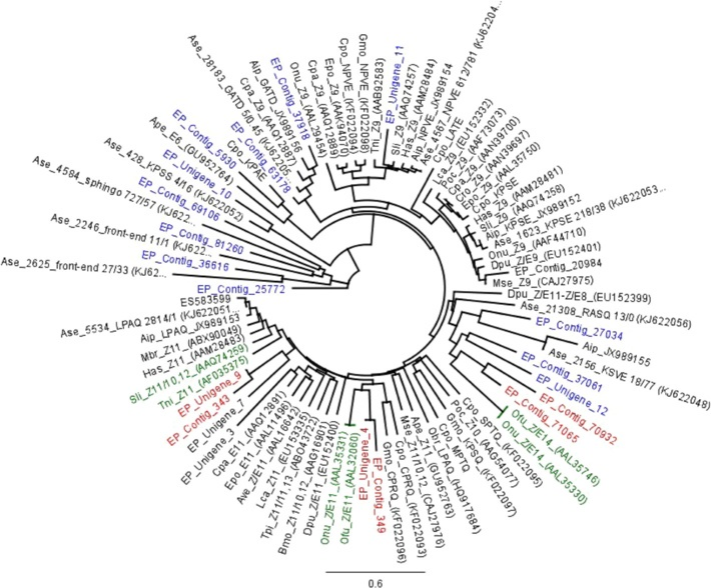
Phylogeny of Lepidopteran desaturase genes

The phylogenetic analysis of *E. cautella* desaturases with other moth desaturases is shown in the Fig. 7. Based on the phylogenetic tree, three possible candidate desaturases have been identified, EP_contig_349/EP_unigene_4, EP_unigene_9/EP_contig_343 and EP_contig_70932/EP_contig_71065, which form a clade with ∆9, ∆11, ∆12 and ∆14 desaturases (Fig. 7). EP_contig_349/EP_unigene_4 is closely related to *O. nubilalis* and *O. furnacalis* ∆11 and ∆9 desaturases (GenBank: AAL35331 and AAL320660, respectively). EP_unigene_9/EP_contig_343 is in the clade with *Spodoptera littoralis* desaturases, which have Z9 and E10,12 desaturase activities (GenBank: AAQ74259). The third putative desaturase type includes EP_contig_70932 and EP_contig_71065 and is closely related to the ∆14 desaturases of *O. furnacalis* and *O. nubilalis* (GenBank: AAL35746 and AAL35330, respectively) (Fig. 7). The one and only ∆14 desaturase reported so far is from a Pyraloidea moth, *O. furnacalis* [18, 57], although a later study showed several cryptic ∆11- and ∆14-desaturase genes exist in the *O. nubilalis* genome [59]. Further studies on the functional expression of desaturases in *E. cautella* will provide more insights into the origin and evolution of the ∆14 desaturases.

### β-oxidation enzymes

Once the ∆14 desaturase introduces a double bond in palmitate it forms *E*14-16:acid (Fig. 3), which is later subjected to chain shortening by β-oxidation, resulting in the fatty acyl pheromone precursor, *E*12-14:acid (Fig. 3). In the alternative pathway, it is involved in the 16:acyl chain being shortened to 14:acyl by β-oxidation. β-oxidation is the action of a series of enzymes, working sequentially and forming a reaction spiral [35].

First, by the action of acyl CoA oxidase (ACO) (in peroxisomes) and acyl-CoA dehydrogenase (ACD) (in mitochondria), acyl-CoA is converted into E2-enoyl-CoA. There was an earlier report of four different ACDs, short-chain, medium-chain, long-chain and very-long-chain ACDs, depending on the fatty acyl chain-length specificities [60]. However, there is no report characterizing the ACDs involved in moth pheromone biosynthesis. It is possible that medium-chain ACDs could be more active because they act on hexanoyl-CoA, whereas long-chain ACDs preferentially act on octanoyl-CoA and longer chain-length substrates. We found many candidate genes of ACDs and acyl-CoA oxidases in the PG of *E. cautella.* In particular, EP_unigene_3_ACD [EC:1.1.1.211] and EP_unigene_2_ACD are the most abundant ACDs (271 and 219 RPKMs, respectively), and EP_unigene_1_ACO [EC:1.3.3.6] and EP_unigene_6_ACO are the most abundant acyl-CoA oxidases in the PG (192 and 127 RPKMs, respectively) (Additional file 8: Table S7). Moreover, we found two unigenes (EP_unigene_9_ACD [EC:1.3.3.6] and EP_contig_419_ACD) of isovaleryl coenzyme A dehydrogenase, which is specific to the metabolism of branched-chain fatty acids [61] (Additional file 8: Table S7).

The next step of β-oxidation involves E2-enoyl-CoA, which is reversibly hydrated by enoyl-CoA hydratase to L-3-hydroxylacyl-CoA. Two kinds of enoyl-CoA hydratases have been identified in mitochondria, one specialized for crotonyl-CoA (4C) and the other one being a long-chain enoyl-CoA hydratase, which effectively hydrates medium and long-chain substrates [62]. We found many candidate genes for enoyl-CoA hydratases and, among these, the EP_unigene_4_ECH [EC:4.2.1.17] is the most abundantly expressed in the PG of *E. cautella* (RPKM: 323). It shows a 93 % amino acid identity with the PG of *Papilio xuthus* (GenBank: BAM18079) (Additional file 8: Table S7).

The third reaction involves a reversible dehydrogenation of L-3-hydroxyacyl-CoA to 3-ketoacyl-CoA catalyzed by L-3-hydroxyacyl-CoA dehydrogenase. There are three different kinds of L-3-hydroxyacyl-CoA dehydrogenases that have been reported in mitochondria, long-chain, medium-chain and short-chain L-3-hydroxyacyl-CoA dehydrogenase (active with long-, medium- and short-chain substrates, respectively) [55, 62]. In the *E. cautella* PG, we found many candidate genes for long-, medium- and short-chain L-3-hydroxyacyl-CoA- dehydrogenases. EP_unigene_4_HCD [EC:1.1.1.35] is highly abundant in the PG (RPKM: 542), followed by EP-Unigene_2_HCD (RPKM: 271), and they both show high amino acid identities with *D. plexippus* and *B. mori* hydroxyacyl-CoA-dehydrogenases (GenBank: EHJ72407 and NP_001040132, respectively) (Additional file 8: Table S7).

Finally, 3-ketoacyl-CoA is cleaved by a thiolase between its α- and β-carbon atoms, producing the two carbon shorter substrate (*E*14-16:acid to *E*12-14:acid or C16: acid to C14: acid). Three kinds of thiolases exist in mitochondria, acetoacetyl-CoA thiolase or acetyl-CoA acetyltransferase (specific to acetoacetyl-CoA), 3-ketoacyl-CoA thiolase or acetyl-CoA acyl transferase (acts on C4-C16 unsaturated fatty acids), and long chain 3-ketoacyl-CoA thiolase (component enzyme of the membrane-bound tri-functional β-oxidation), where the first two kinds of thiolases are components of the soluble matrix enzyme complex [63]. We found eight candidate thiolase genes in the PG of *E. cautella*, with EP_contig_2704_KCT [EC:2.3.1.16] having the most highly abundant transcript in the PG (RPKM: 108) (Additional file 8: Table S7) and a 94 % amino acid identity with *D. plexippus* (Additional file 8: Table S7) (GenBank: EHJ7447).

The degradation of unsaturated fatty acids requires auxiliary enzymes, such as delta-3, delta-2 trans-enoyl-CoA isomerase and 2,4-dienoyl-CoA reductase, to modify the structure of the double bond during the β-oxidation process [64]. In the *E. cautella* PG, we found four delta-3, delta-2 trans-enoyl-CoA isomerases, two mitochondrial and two peroxisomal, and among these EP_unigene_1_TECI [EC:5.3.3.8] has the most abundant transcripts (RPKM: 177) (Additional file 8: Table S7). Additionally, we found a delta(3,5)-delta(2,4)-dienoyl-CoA isomerase [EC:5.3.3.-], which is specialized for processing odd-numbered double bonds [61] (Additional file 8: Table S7).

Moth pheromones generally consist of 10C-16C compounds synthesized from C16-C18 fatty acid moieties, involving many chain-shortening reactions [44]. Previous research has mostly been related to the desaturases and functional group modification enzymes, while research on the chain-shortening enzymes involved in pheromone biosynthesis has been meager. In the present study, we found many promising candidates that may be involved in β-oxidation, and further research on their heterologous expression, or RNAi, could reveal their significance in *E. cautella* pheromone biosynthesis.

### Fatty Acyl Reductase (FAR) [EC:1.2.1.-]

FAR enzymes catalyze the reduction of fatty acyl precursors to fatty alcohols in a reaction that is dependent upon NADPH as a cofactor [19, 22–24]. FAR genes have been shown to function in pheromone biosynthesis in moth species directly through the production of an alcohol that confers species specificity or indirectly through the biosynthesis of precursor compounds [10, 21]. The number of FAR genes per genome can vary greatly between organisms. In vertebrates, there are two reductase genes present in the genomes, whereas there are more than a dozen present in the moth *O. scapulalis* [22]. The FAR gene family undergoes birth- and death-related evolution [65]. Even though the evolutionary origins of this gene family are not well understood, it has been assumed, based on protein sequence similarity, that the acyl-CoA synthetase, acyltransferase and oxidoreductase gene families are close relatives of this family, and thus form a superfamily [65]. In the *E. cautella* PG transcriptome pooled data, we identified 28 FAR-like genes, which included partial and full-length sequences (7), and BLASTx results identified them as putative FAR-like genes (Table 3). Based on the sequence assembly, multiple sequence alignment and BLASTx hit results, we named them uniquely; however, they may represent partial sequences of the same FARs. We took care to avoid duplications; however, if the full-length sequence was not available in our transcriptome dataset or in the NCBI database, then it could be present in partial sequences. EP_unigene _9 (2,019 bp) is the most highly expressed FAR in *E. cautella*’s PG (RPKM: 772). It showed a 67.6 % identity with *D. plexippus* (GenBank: EHJ72233), followed by EP_unigene _1 (RPKM: 742) and EP_unigene _14 (RPKM: 536). It shares a 77.3 % amino acid identity with *O. nubilalis* and a 59.1 % with *D. plexippus* (GenBank: ADI82778 and XP_004926010, respectively). All other FARs, except two, EP_unigene_2 and EP_unigene_3 (502 and 487 RPKMs, respectively), have a low abundance, with RPKM values of less than 100, in the PG transcriptome (Table 3). Further studies on the tissue specificity of each FAR to determine the PG-specific FARs is in progress.

**Fig. 8:**
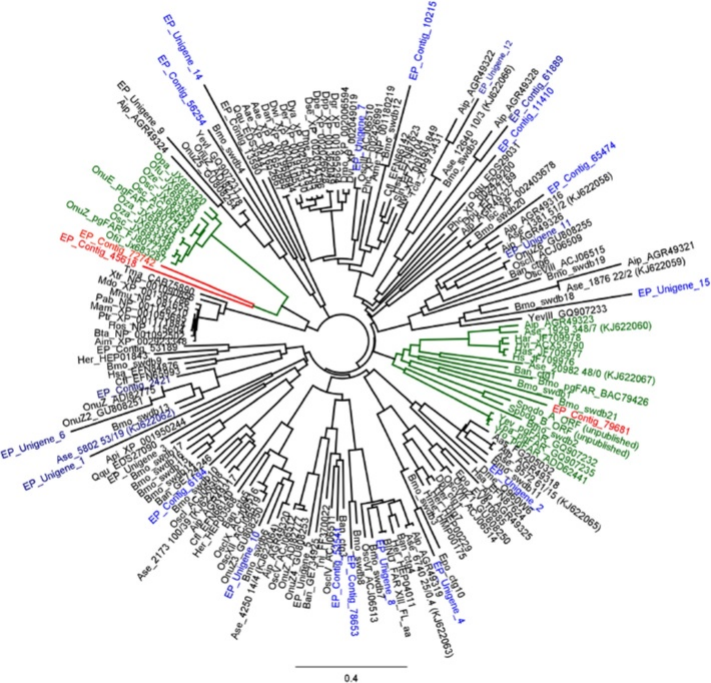
Phylogeny of Lepidopteran fatty acyl reductase (FAR) genes

The phylogenetic analysis of moth FARs is shown in Fig. 8. Based on the phylogenetic tree, three possible candidate FARs were identified, EP_contig_72742, EP_contig_45618 and EP_contig_79681, which formed a clade with moth pgFAR (Fig. 8). Until now pgFAR had been characterized from *B. mori* [19], *O. scapulalis* [22], nine *Ostrinia* spp. [20, 21], three *Yponomeuta* spp. [23], as well as *Helicoverpa* and *Heliothis* [24]. In the *E. cautella* PG, EP_contig_72742 and EP_contig_45618 FARs formed a cluster with the *Ostrinia* pgFARs (Fig. 8), and EP_contig_79681 formed a cluster with the *Yponomeuta*, *Helicoverpa* and *Heliothis* pgFAR clade (Fig. 8). Further studies on tissue-specific expression and heterologous gene expression in a yeast system are in progress.

**Fig. 9:**
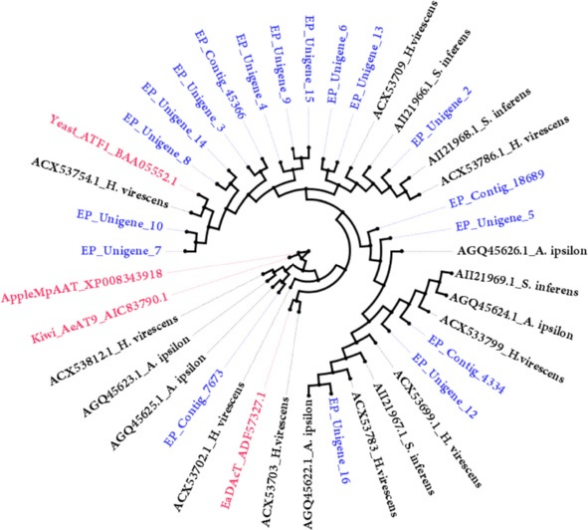
Maximum Likelihood (ML) tree of the Fatty Acetyltransferase (FAT) from the *E. cautella* PG and various FATs from the *A. ipsilon* and *H. virescens* PGs

### Aldehyde Reductase (AR) [EC:1.1.1.-]

ARs are members of the aldo-keto reductase superfamily and can reduce long-chain acyl-CoA to form aldehyde intermediates [44]. In insects that use both an alcohol and an aldehyde as part of their pheromone, it is unclear how the production of both components occurs. Even though we did not identify any aldehyde precursors in the PG of *E. cautella* during the FAME analysis, we are still discussing ARs in this study because ARs and FARs share an evolutionary history, and the gene families are closely related [65]. In the *E. cautella* PG we identified 11 transcripts with homology to the aldo-keto reductases of *A. ipsilon*, *Papilio xanthus*, *B. mori*, *Chilo suppressalis* and *D. plexippus* (Table 3). The derived protein sequences of these 11 transcripts showed a 68–82 % amino acid identity with their homologs in other insects. All of the AR transcripts were present at a low abundance (less than 50 RPKM) in the PG transcriptome (Table 3); therefore, we assumed that AR does not have a role in *E. cautella* pheromone biosynthesis.

### Fatty Acetyltransferases (FAT) [EC:2.3.1.-]

To produce the acetate ester pheromone components, most moths use an acetyl-CoA: fatty alcohol acetyltransferase that converts fatty alcohols to acetate esters [2–5]. The genes involved in this step have not been characterized from any insects [2–5, 10]. However, different acetyltransferases, which have a characteristic motif (HXXXD) and a conserved region (DFGWG), have been cloned from plants [66]. In the *E .cautella* PG transcriptome, 18 FAT-like genes were identified, showing homology to *D. plexippus*, *B. mori*, *O. scapulalis* and *A. ipsilon* (Table 3). Except for two FATs, they showed a greater than 70 % amino acid identity, the highest being EP_contig_7673 at 95 %, with *A. ipsilon* (GenBank: AGQ45625). We searched the conserved domains (CD) within the protein or coding nucleotide sequence databases at NCBI, but none of the *E. cautella* PG acetyltransferases belonged to the plant category FATs, suggesting that *E. cautella* may not express this gene family or that they have undergone substantial evolutionary changes. Nevertheless, most of the *E. cautella* FATs had hits to members of the N-acyltransferase (NAT) superfamily with CD accession no. cl17182 (Table 3). The CD accession nos. of *E. cautella* PG acetyltransferases are given in the Table 3 (fatty acetyltransferase). All FAT transcripts were present at low abundance levels (less than 120 RPKM) in the *E. cautella* PG transcriptome (Table 3); therefore, we could not predict which, if any, had roles in pheromone biosynthesis. However, phylogenetic analysis showed that EP_unigene_7 and EP_unigene_10 clustered with yeast (*S. cerevisiae*) alcohol acetyltransferase [EC:2.3.1.84] (GenBank: BAA05552.1), which catalyzes the esterification of isoamyl alcohol by acetyl coenzyme A (Fig. 9). There are several candidate *E. cautella* FAT transcripts (EP_unigene 3, 4, 6, 8, 9, 13, 14 and 15, and EP_contig 45366) that did not form a clade with any other FAT transcripts of the *A. ipsilon* or *S. inferens* transcriptome datasets (Fig. 9).

### Candidate pheromone degrading enzymes in the *E. cautella* PG

Pheromone molecules would be potentially harmful to insects if they remained on the ORs after they had stimulated the ORNs. Many studies emphasize that there are mechanisms to protect the ORNs using ODEs [67], including esterases [67–69], aldehyde oxidases [70–72], cytochrome P450 [73–75], carboxyl esterases (cxe) [67] and glutathione S-transferase (GST) [76], which occur in major chemosensory tissues, including the terminal abdominal segment [77]. In general, the esterase gene family consists of three major groups: intracellular (highly expressed in antenna and involved in detoxification), neuro/developmental (neural tissues in antennae) and secreted esterases (expressed in different tissues and associated with specific hormonal and pheromonal functions) [32]. The secreted esterase class contains five major subclasses (glutactin, juvenile hormone (JH) esterases, JHEs-like enzymes, β*-*esterases and semiochemical esterases), and the ODEs are members of the semiochemical esterases, which are potentially involved in the degradation of pheromone compounds and plant volatiles [32]. The secreted esterase are of three different types, antennal enriched (ODEs in antenna), both antennal and PG-enriched (pheromone degradation) and esterases expressed throughout the body (not pheromone specific) [32].

**Fig. 10:**
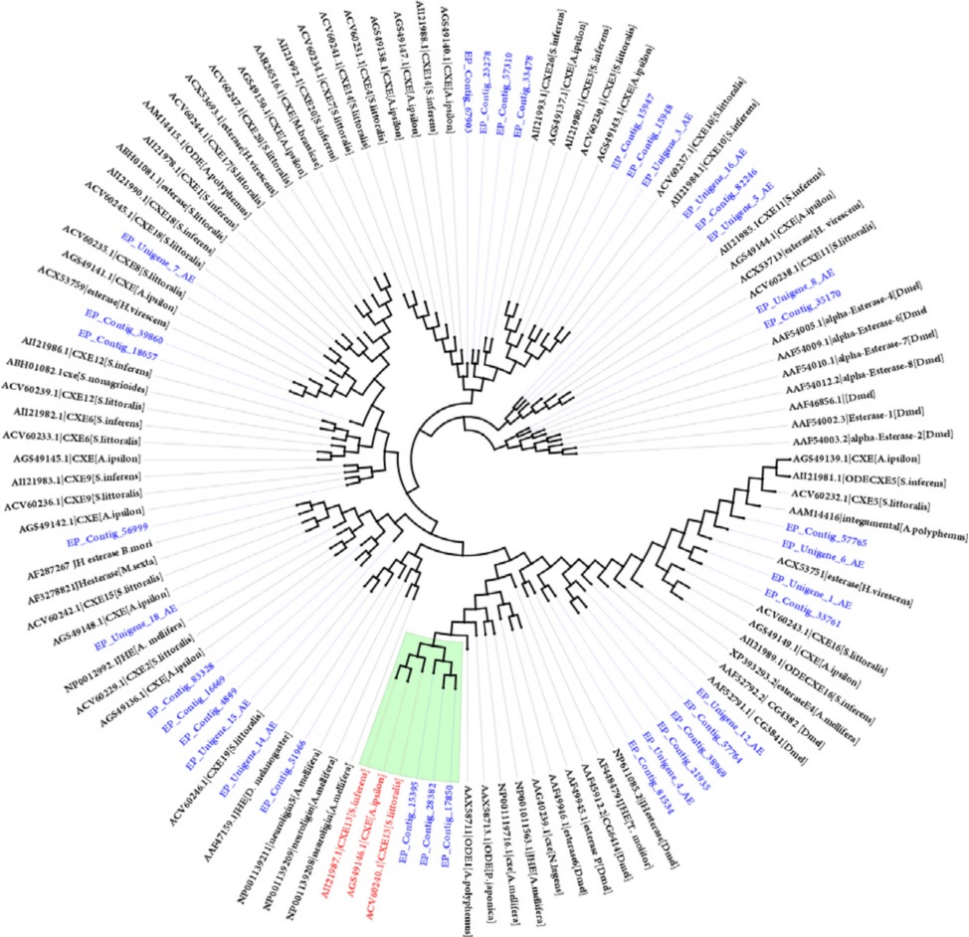
Maximum likelihood (ML) tree of insect esterases (cxes)

In the present study, we identified 36 transcripts predicted to encode esterases in the *E. cautella* PG, and the BLASTx results showed that they shared very high amino acid identities with the esterases of *S. exigua*, *S. littoralis*, *B. mori* and *D. plexippus* (Additional file 9: Table S8). By comparing *E. cautella* cxes with *S. littoralis* [67, 78] and *S. inferens* [40] we identified the *E. cautella* cxes that are known to be both antennal and PG-enriched, including cxe 4, cxe5, cxe10, cxe11, cxe13 and cxe16 (Fig. 10). All of the esterase transcripts were present at low abundance levels (less than 80 RPKM) in the PG transcriptome (Additional file 9: Table S8); however, cxe13 (EP_contig_15395_AE, EP_contig_17850_AE and EP_contig_ 28382_AE) had the most highly expressed esterase transcript level (RPKM: 58) (Additional file 9: Table S8). Durand et al. [78] reported the ubiquitous expression of cxe13 in *S. littoralis* with a specific role in pheromone processing. Homologous cxe13s also reported in *Antheraea polyphemus* and *Popilia japonica* were found to degrade the pheromone *in vitro* [68]. Thus, we assumed that cxe13 has a specific role in *E. cautella* pheromone processing and degradation.

To assign putative functions and correct identifications, an esterase phylogenetic tree was constructed using 36 *E. cautella* transcripts and other insect (*Drosophila melanogaster*, *Apis mellifera*, *A. polyphemus*, *B. mori, S. lottoralis*, *A. ipsilon* and *S. inferens*) esterases (Fig. 10). The phylogeny showed that cxe10 (EP_Unigen_10), cxe11 (EP_Unigen_5, EP_Unigen_16 and EP_contig_82246), cxe13 (EP_contig_15395, EP_contig_17850 and EP_contig_ 28382), cxe18 (EP_Unigen_7), cxe14 (EP_contig_67903), cxe26 (EP_contig_33478) and cxe19 (EP_Unigen_14) clustered with the corresponding cxes of *S. littoralis* [78] and *S. inferens* [40] (Fig. 10). The phylogeny also revealed that EP_contig_38969, EP_contig_57765 and EP_contig_51966 clustered with the JH esterase of *D. melanogaster* (ACZ94438), integumental esterase of *A. polyphemus* (AAM14416) and neuroligins of *A. mellifera* (NP_001139211), respectively (Fig. 10)*.*

### Candidate pheromone carrier proteins in the *E. cautella* PG

In insects, the odorant binding proteins and the chemosensory proteins are involved in olfaction and contact chemosensation [79, 80]. Specific OBPs that are involved in pheromone binding and transport are called PBPs [26, 79]. In moths, OBPs are divided into three main classes based on sequence alignment and their localization in the insect body. PBPs are preferentially expressed in pheromone-sensitive sensilla trichodea from male antennae, GOBPs are mainly found in the female antennae, in particular in the plant odor sensitive sensilla basiconica [81–83] and ABPXs represent the third class [84]. Based on the sensillar distribution, PBPs may be involved in pheromone binding, GOBPs in binding the plant volatiles and ABPXs in binding general odorants. The functions of OBPs are to solubilize the hydrophobic odorant molecules in the aqueous lymph surrounding the dendrites and to protect them from the ‘degrading esterases’ circulating in the lymph [85]. Additionally, the OBPs deliver the odorant stimuli molecules to specific ORs by releasing the odorant upon contact with membrane structures [86].

Although CSPs are expressed all over the insect’s body, they exist mainly in the legs and in contact chemosensory sensilla. CSPs consist of polypeptide chains of about 110 amino acids with a molecular weight of 12–13 kDa. OBPs have six highly conserved cysteines, whereas CSPs have only four cysteines [79]. Several studies have shown that moth sex pheromones are protected against degradation until they are released from the female PG, and it has been proposed that OBPs and CSPs participate in this process [87, 88].

**Fig. 11:**
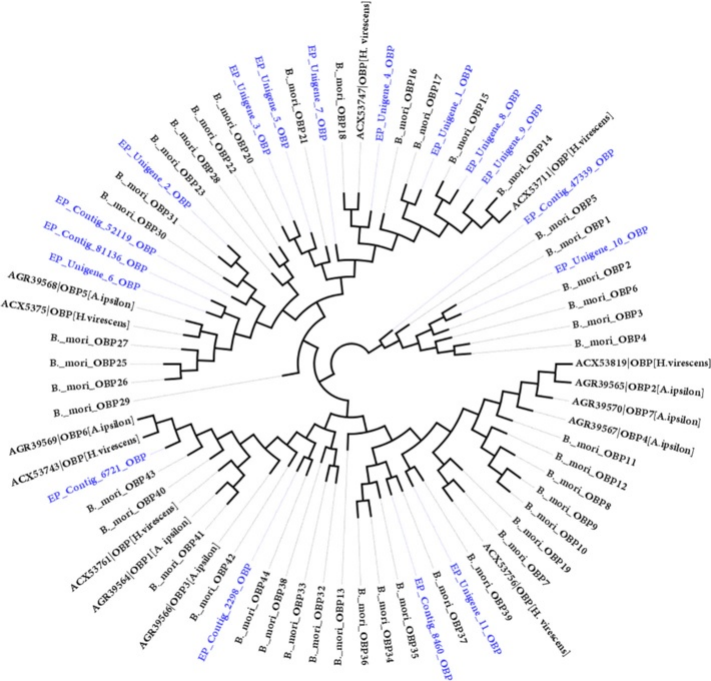
Maximum Likelihood (ML) tree of the Odorant Binding Proteins (OBPs)

In the *E. cautella* PG, we identified transcripts of 7 CSPs and 17 OBPs (Additional file 10: Table S9), all containing the typical insect OBP [88, 89] or CSP sequence motifs [87], respectively. One CSP transcript, EP_unigene_4_CSP, appears to be expressed at an extremely high level (cumulative RPKM: 148,880) in the PG and has a relatively high abundance of transcripts in the PG transcriptome (Additional file 10: Table S9). Phylogenetic analysis shows EP_unigene_4_CSP clustered with *B. mori* CSP5 (*Bmor*CSP5) [89], *H. virescens* CSP [38] and *A. ipsilon* CSP8 (*Aips*CSP8) [39] (Additional file 12: Figure S11). *Aips*CSP8 shows a high expression level in the PG and has extremely abundant transcripts in the *A. ipsilon* PG [39]. Previously, RNAi studies suggested a novel role for a CSP5 in the development of the embryonic integument in *A. mellifera* and were found to be highly expressed in the ovary [90]. All OBPs have very low expression levels in the PG, but EP_contig_6721_OBP and EP_contig_8460_OBP were comparatively highly expressed OBP transcripts (RPKM: 103) (Additional file 10: Table S9). To assign putative identifications, an OBP phylogenetic tree was constructed with *E. cautella* OBP transcripts and the *B. mori* OBPs [91] (Fig. 11). The phylogeny identified *E. cautella* OBPs homologous to *B. mori* OBP39 (EP_unigene_11_OBP), OBP37 (EP_contig_8460), OBP44 (EP_contig_2298), OBP43 (EP_contig_6721), OBP31 (EP_unigene_2), OBP20 (EP_unigene_3), OBP18 (EP_unigene_4), OBP17 (EP_unigene_1), OBP15 (EP_unigene_8), OBP14 (EP_unigene_9) and OBP1 (EP_unigene_10) (Fig. 11). A six-cysteine signature is the most typical feature of classical OBPs [88, 89] and *E. cautella* OBPs carry most of the conserved cysteine residues (data not shown). The *B. mori* OBPs reported above were found to express in multiple tissues including the terminal abdominal segments (ovary, hind gut, fat body, Malpighian tubule and PG) [91]. Further studies are needed to clarify the roles of the CSPs and OBPs in protecting against the degradation of the *E. cautella* pheromone prior to its release from the female PG.

We found very low expression levels of three ABP transcripts (less than 60 RPKM) and two PBP transcripts (less than 20 RPKM) in the *E. cautella* PG (Additional file 10: Table S9). Widmayer et al. [77] detected PBP2 in the *H. virescens* ovipositor tip, and we found a homologous sequence in the *E. cautella* PG (EP_contig_73451_PBP; hereinafter EcauPBP2) showing an 89.7 % amino acid identity with *A. transitella* (ACX47890). In *H. virescens*, the response to the major pheromone component (*Z*11-16: Al) is mediated by the PBP2 and the pheromone receptor HR13 [77].

The expression of odorant receptor proteins has been shown to be necessary and sufficient for odor detection in insects [25]. Widmayer et al. [77] detected the pheromone receptors HR2, HR6 and HR13 in the *H. virescens* ovipositor tip, and HR13 along with PBP2 mediated abdominal responses to the emitted pheromones. In the *E. cautella* PG, we identified 21 putative **OR**s, 3 candidate IRs and 2 candidate SNMPs (Additional file 11: Table S10). To assign putative identifications, an **OR** phylogenetic tree was constructed using *E. cautella***OR** transcripts, *H. virescens* HR2, HR6 and HR13 [77] and *H. armigera***OR**s [92] (Additional file 13: Figure S12). Based on the phylogenetic analysis, EP_unigene_5_**OR** (hereinafter EcauOR13) is closely related to the pheromone receptor of *H. virescens* H13 [77] and OR13 of *H. armigera* (HarmOR13) [92] (Additional file 13: Figure S12). Recently, Liu et al., [92] reported HarmOR13 responds to the *H. armigera* pheromone compound (*Z*11-16: Ald) using calcium imaging studies, and they also found significant gene expression levels in the terminal abdominal segments (TASs) of *H. armigera*. Hence, we assume that in the *E. cautella* PG, the response to the pheromone component (*Z*9,*E*12*-*14:OAc) is mediated by the pheromone binding protein, EcauPBP2 and the receptor type EcauOR13, and it may have an important role in the transport and release of the pheromone molecule. Further studies on the functional characterization of EcauPBP2 and the receptor type EcauOR13 will be necessary to prove the hypothesis. Phylogenetic comparisons of *E. cautella***OR**s with those of *H. armigera* [92] identified the *E. cautella* receptor proteins expressed in the terminal abdominal segment (Additional file 13: Figure S12). All the receptor protein transcripts were present in very low abundance (less than 50 RPKM) in the PG (Additional file 11: Table S10). It is noteworthy that a SNMP, EP_contig_ 1371_SNMP, which has a greater than 70 % amino acid identity with an *O. nubilalis* SNMP (GenBank: ADQ73889), was expressed highly in the TAS (RPKM: 102) (Additional file 11: Table S10). Further studies on these PG-expressed ORs, SNMPs and IRs involved in *E. cautella* pheromone binding and transport need to be performed.

## Conclusions

The tropical warehouse moth, *E. cautella*, listed as a major storage pest, is a serious threat to the date and chocolate factories of the Middle East and Europe. Our study provides comprehensive information on the pheromone molecules, **biosynthetic pathways** and genes expressed in the PG that are related to pheromone biosynthesis, degradation, transport and release. Our study provides information on the *E. cautella* sex pheromone and precursors in the PG, and shows two possible pheromone **biosynthetic pathways**. Both pathways initiate from C16:acyl-CoA, and one involves ∆14 and ∆9 desaturation to generate *Z*9,*E*12-14:acyl, while the other pathway involves the chain shortening of C16:acyl-CoA to C14:acyl-CoA, followed by ∆12 and ∆9 desaturation to generate *Z*9*,E*12-14:acyl-CoA. Finally, reduction and acetylation generate *Z*9,*E*12-14:OAc. Using the Illumina sequencing of the PG transcriptome, we identified candidate genes: PBAN receptor isoforms A and C, FATPs, ACCs, FASs, DESs, several β-oxidation enzymes, FARs and FATs. Two transcripts, EP_unigene_9_1286/EP_contig_343 and EP_contig_349/EP_contig_145 are highly expressed, form a cluster with moth desaturases and might be involved in the ∆14 or ∆12 and ∆9 desaturation processes. The highly expressed β-oxidation enzymes, dehydrogenases, oxidases, hydratases, thiolases, enoyl and dienoyl isomerases, which are involved in the chain shortening, have been identified. Three possible candidate FARs have also been identified, EP_contig_72742, EP_contig_45618 and EP_contig_79681, which form a cluster with moth pgFAR, and thus, might be involved in the reduction step of *Z*9,*E*12-14:acid to *Z*9,*E*12-14: alcohol. Two possible FATs, EP_unigene_7 and 10, which clustered with yeast alcohol acetyltransferases, are good candidates for gene expression studies. We found many promising candidate PBEs, and further research using heterologous gene expression or RNAi could reveal the significance of these genes in *E. cautella* pheromone biosynthesis. Several candidate esterases have also been identified, including cxe13 (contig 15395; 17850 and 28382), which may be involved in signal inactivation by removing the pheromone molecules. The CSP (EP_unigene_4) is the most highly abundant transcript of the *E. cautella* PG, and, together with two OBPs (Contig 6721 and 8460) and one EcauPBP2 (EP_contig_73451), it may have an important functional role in protecting sex pheromones from the activities of esterases, as well as in the transport and release of the pheromone molecules. The ORs, EcauOR13 (EP_unigene_5) and EcauPBP2, meditate abdominal responses to the emitted pheromone in *E. cautella*, and may have important roles in the transport and release of the pheromone molecules. Our study provides strong background information on the enzymes involved in pheromone biosynthesis that will be useful for the *in vitro* production of *E. cautella* sex pheromones. The study also provides information on novel genes involved in the transport, release and degradation of pheromone compounds, increases the understanding of the sex pheromone detection system, and it may provide potential targets for disrupting the pheromone-based communication system in *E. cautella* for control purposes.

## Methods

### Chemicals

C14:COOMe and C16:COOMe were purchased from Sigma. (*Z,E*)-9,12- tetradecadienyl acetate was purchased from Pherobank. *E*11-13:OH, *E*14-16:COOMe, *Z*9-14:COOMe, *E*12-14:COOMe, *Z*9-16:COOMe, *E*9-16:COOMe, *Z*11-16:COOMe, *E*11-16:COOMe and *Z*9*E*12-14:COOMe were purchased from Pest Control of India Private Limited (Mumbai, India). All standard compounds were of > 98.0 % purity and diluted in *n*-hexane (250 ng/μl).

### Insects

The *E. cautella* individuals (dried date fruit strain) were originally collected from the Al Hasa date factory (Saudi Arabia) (25°38′ N, 49°60′ E), and were established on an artificial diet comprised of dried broken wheat, peptone and sucrose as the main components. Pupae were sexed, and the female pupae were placed in cages at 24 ± 2.0 °C under a L16:D8 photoperiod. The female pupae were collected separately, and the newly emerged adults were maintained in a vial under the same conditions.

### Sex PG extraction and fatty-acyl precursor analysis

The terminal abdominal segments (TASs) (segments 8–10) of individual virgin *E. cautella* one day before adult eclosion, and of 0-, 1-, 2-, and 3-day-old female moths at mid-scotophase, were excised with micro-scissors. Each gland was extracted for 30 min at room temperature (RT) in a glass insert vial containing 50 μL *n*-hexane (Sigma) and 250 ng/μL of *E*11-13:OH as an internal standard (IS). The individual PG extracts were stored at −20 °C until GC-MS analysis.

Total lipid and residue extracted from *E. cautella* PG were subjected to base methanolysis to convert fatty acyl moieties to the corresponding methyl esters [93]. Two glands were homogenized with a glass rod and transferred into a conical glass vial, and the lipid content was extracted in 500 μL methanol: chloroform (1: 2, v: v), vortexed vigorously for a few minutes and incubated at RT for 30 min. Later, the organic phase was transferred into a new glass tube, and the solvent was evaporated under a gentle stream of nitrogen. Then, 1 mL of 2 % H_2_SO_4_ (in methanol) was added and incubated at 90 °C for 1 h. Later, 1 mL of milliQ water and 1 mL of *n*-hexane were added and vortexed vigorously for a few seconds. The upper hexane portions were then transferred into a new glass vial and stored at −20 °C prior to GC-MS analysis.

Both pheromone extracts and the methyl ester samples were subjected to GC-MS analysis on a Agilent 7850A GC coupled to a mass detector (Agilent 5975C) and equipped with a medium-polar INNOWax column (100 % polyethylene glycol, 30 × 0.25 mm I.D., film thickness 0.25 mm, Agilent Technologies, USA). The GC-MS was operated in electron impact mode (70 eV), the injector was configured in split-less mode at 220 °C, and helium was used as carrier gas (velocity: 30 cm/s). The oven temperature was set to 80 °C for 1 min, then increased at a rate of 10 °C/min up to 210 °C, followed by a hold at 210 °C for 15 min, and then increased at a rate of 10 °C/min up to 230 °C, followed by a hold at 230 °C for 20 min.

### RNA isolation, cDNA synthesis and library construction

The TASs of ~100 virgin 2- to 3-day-old female *E. cautella* moths at mid-scotophase were excised. The total RNA of *E. cautella* PGs was prepared using a NORGEN purification kit (NORGEN Biotek Corp., Canada). Purification is based on spin column chromatography using Norgen’s proprietary resin as the separation matrix, and the procedures were performed according to the manufacturer’s instructions. The quantity and quality of the total RNA was validated using Qubit® 2.0 Fluorometer (Invitrogen, Life Technologies), and the RNA integrity was further confirmed using the 2100 Bioanalyzer (Agilent Technologies) with a minimum RNA integrated number value of 6.8.

The paired-end cDNA libraries were prepared using Illumina protocols and sequenced on the Illumina HiSeq platform. Briefly, the cDNA library was constructed using a TruSeq™ RNA Kit (Illumina Inc.), which consists of mRNA purification and fragmentation from total RNA, synthesizing first and second strands of cDNA, performing cDNA end repair and adenelylating the 3′ ends, followed by adapter ligation and cDNA fragment enrichment. These products were purified and enriched using PCR to create the final cDNA library. Finally, the cDNA library quantity was validated using a Qubit® 2.0 Fluorometer (Invitrogen, Life Technologies), while the quality was validated using an Agilent Technologies 2100 Bioanalyzer prior to the HiSeq Illumina sequencing.

### Illumina sequencing

HiSeq Illumina sequencing was performed at the core sequencing facility of the King Abdulla University of Science and Technology (KAUST), Jeddah, Saudi Arabia. The insert size of the library was ~306 bp. Image deconvolution and quality value calculations were performed using the Illumina GAPipeline1.3. All sequencing reads were submitted to the SRA of NCBI under the accession number SRX646348.

### Sequence pre-processing, assembly and analysis

A quality control step was first performed on raw sequencing reads using the NGS QC Toolkit [94]. Standard RNA adapter sequences and regions of poor quality were clipped using the CLC Genomic Server and its tool ‘Trim Sequences’. The *de novo* assembly was performed by the CLC Genomics Server using the scaffolding option and the mapping reads back to transcripts option. This Transcriptome Shotgun Assembly project has been deposited at DDBJ/EMBL/GenBank under the accession GBXH00000000. The resulting *de novo* assembled transcripts were locally searched against the non-redundant (*nr*) protein database using the BLASTx algorithm (e ≤ 0.001) implemented in the standalone version of the blast + tool [95] and stored in the BLAST archive format (ASN.1). Later, the results were parsed into the required format (XML, tabular, pairwise) using the blast_formatter tool. XML BLASTx results were imported into the BLAST2GO annotation tool. The RPKM values were calculated for assembled transcripts based on their mapping data according to the formula published by Mortazavi et al. [96].

### Gene identification and functional annotation

Following the assembly, each transcript was identified by local or web-based searches using the BLASTx and BLASTn programs of NCBI [97]. Blast hits with e-values less than 1.0E-^5^ were considered as significant [98], and the genes were putatively assigned to each contig based on the BLASTx hit with the highest score value. The BLAST XML files were uploaded to BLAST2GO and the mapping, gene annotation, INTERPRO and KEGG analyses were performed as with BLAST2GO [99, 100]. Each gene was checked in terms of molecular function, biological process or cellular component.

Transcripts containing errors leading to misassemblies were edited using Geneious v7.1.5 (www.geneious.com/), *de novo* assemblies of isotigs were performed and the open reading frame (ORF) of each unigene was determined using the ORF finder tool (NCBI). INTERPRO analysis terms were assigned by BLAST2GO [101] through a search of the *nr* databases. To annotate the pooled assembled transcriptome, we performed a BLAST search against the *nr* databases of NCBI, UniProtKB and KEGG using an e-value cut-off of 1.0E5.

### Comparative analysis of PG transcriptome

The *A. ipsilon* PG transcriptome data were downloaded from NCBI (SRX189143) and assembled in the CLC Genomics Server. The *H. virescens* PG ESTs (14,112 with accession numbers: GR958232-GR972305 and GT067784-GT067747 [38], and the *B. mori* PG ESTs (10,501 with accession number: BP184340-BP182009, AV404455-AV403746 and DC552314-DC544856) were downloaded from the dbEST database of NCBI (http://www.ncbi.nlm.nih.gov/nucest) and saved as FASTA files. The comparative analyses of *E. cautella*, *A. ipsilon* [39], *H. virescens* [38] and *B. mori* [39] PG transcripts were performed based on the best bidirectional hit results (first 10 blast hits) (reciprocal BLASTn, e-value less than 1.0E − 6).

### Identification of candidate genes involved in *E. cautella* pheromone biosynthesis

The search for PBEs in our NGS dataset was based on the candidate genes involved in the pheromone biosynthesis in *B. mori*. We focused on the following target genes: PBANs, ACCs, FASs, desaturases, β-oxidation enzymes, FARs and FATs.

### Identification of putative genes involved in fatty acid transport and pheromone degradation

A fatty acid transport protein, BmFATP, was identified from the PG of the silkworm *B. mori*, which produces a Type-I sex pheromone (bombykol) [47]. BmFATP was shown to facilitate the uptake of extracellular fatty acids into PG cells for the synthesis of bombykol. We performed BLASTx and BLASTn searches to identify *E. cautella* FATP (EcFATP) genes in the *E. cautella* PG NGS dataset.

There were earlier reports that esterases may play a major role in pheromone degradation [67–69]. Therefore, we performed BLASTx and BLASTn searches to identify candidate esterase genes in the *E. cautella* PG assembled NGS dataset.

### Identification of putative genes involved in pheromone transport

Genes encoding OBPs and CSPs were identified through BLASTx and BLASTn searches, as well as by the “OBP sequence motif” C1-X15-39-C2-X3-C3-X21-44-C4-X7-12-C5-X8-C6 [79, 87–89, 91] and the “CSP sequence motif” C1-X6-8-C2-X16-21-C3-*X*2-C4 [87, 89]. Candidate ORs, IRs and SNMP genes were identified by BLASTx and BLASTn searches. Sequence alignments were performed using the ClustalX program [102].

### Phylogenetic analyses

*E. cautella* desaturase and FAR nucleotide sequences were used as query (BLASTx) in the GenBank database, and the desaturase and FAR sequences from different insect species and their amino acids were retrieved for tree construction. The similarity analyses of DNA and protein sequences and a multiple-sequence alignment were performed using the ClustalX program [102], followed by manual inspection. For the phylogenetic analyses, phylogenetic reconstructions were performed using the Geneious tree builder v7.1.5 (www.geneious.com/). The neighbor-joining algorithm analysis was computed using amino acid sequences (Geneious tree builder, Pam250, Jukes-Cantor and Global alignment).

A dataset of esterase sequences was created by retrieving amino acid sequences from NCBI using BLASTx searches of *E. cautella* PG esterases, and maximum likelihood trees were constructed using MEGA v6.0 [103]. Similarly, acetyltransferase, CSP, OBP and OR sequences were retrieved from the NCBI database, and maximum likelihood trees were constructed using MEGA v6.0 [103]. The NCBI accession number for each gene is provided in the tree.

## Additional files

Additional file 1: Figure S1. Comparison of the *E. cautella* proposed pheromone biosynthetic pathway with those of *Spodoptera exigua* and *S. littoralis*. Additional file 2: Table S2. Comparative summary of *E. cautella, A. ipsilon* [39]*, Grapholita molesta* [41], *H.s virescens* [38] and *B. mori* [38] PG transcriptome sequencing assemblies and annotations. Additional file 3: Figure S3. Characteristics of homology searches of *E. cautella* protein-coding genes against the non-redundant protein sequences (*nr*) at NCBI using BLASTp. Additional file 4: Figure S4. Functional assignment terms to query sequences from the pool of GO terms gathered in the mapping step. Additional file 5: Figure S5. Mapping of *E. cautella* protein-coding genes to GO terms associated to BLASTp hits. Additional file 6:
**KEGG predicted pathways (130).**
Additional file 7: Figure S6. KEGG pathway representing the biosynthesis of unsaturated fatty acids. ∆9 desaturase, β-oxidation enzymes (Acyl-CoA oxidase, Acyl-CoA dehydrogenase, Enoyl-CoA hydratase) activities are shown in the pathway. Additional file 8: Table S7. Putative pheromone biosynthesis-related genes involved in β-oxidation in the *E. cautella* PG. Additional file 9: Table S8 Putative pheromone degrading enzymes in the *E. cautella* PG. Additional file 10: Table S9. Putative pheromone carrier proteins in the *E. cautella* PG. Additional file 11: Table S10 Putative receptor proteins in the *E. cautella* PG. Additional file 12: Figure S11. Maximum likelihood (ML) tree of the chemosensory proteins (CSPs). Additional file 13: Figure S12. Maximum likelihood (ML) tree of the OR proteins.

## Abbreviations

NGS: Next-generation sequencing
EST: Expressed sequenced tag
PG: Pheromone gland, TAS, Terminal abdominal segment
*nr*: Non-redundant protein database
bp: Base pair
CD: Conserved domain
GC-MS: Gas chromatography coupled to mass spectrometry
FAME: Fatty-acid methyl ester
ORF: Open reading frame
*Z*11–13:OH: (*Z*)-11-tridecenol
*Z*9,*E*12–14:OAc: (*Z,E*)-9,12- tetradecadienyl acetate
*E*12–14:acid: (*E*)-12-tetradecenoic acid
*Z*9–16:acid: (*Z*)-9-hexadecenoic acid
*E*9–16:acid: (*E*)-9-hexadecenoic acid
*Z*11–16:acid: (*Z*)-11-hexadecenoic acid
*E*11–16:acid: (*E*)-11-hexadecenoic acid
*E*14–16:acid: (*E*)-14-hexadecenoic acid, (fatty acyls and fatty acid methyl esters are named correspondingly)
RPKM: Read per kilobase per million reads
CSP: Chemosensory protein
OBP: Odorant binding protein
OR: Odorant receptor/olfactory receptor
IR: Ionotropic receptor
SNMP: Sensory neuron membrane protein
ODE: Odorant-degrading enzyme
cxe: Carboxyl esterases
JH: Juvenile hormone
PBP: Pheromone binding protein
PBAN: Pheromone biosynthesis activating neuropeptide
ACC: Acetyl-CoA carboxylase
FAS: Fatty acid synthase
ACD: Acyl CoA dehydrogenase
ACO: Acyl-CoA oxidase
ECH: Enoyl-Co-A hydratase
HCD: L-3-hydroxyacyl-coenzyme A dehydrogenase
KCA: 3-ketoacyl CoA-thiolase
TECI: delta-3, delta-2 trans enoyl CoA Isomerase
DECI: delta(3,5)-Delta(2,4)-dienoyl-CoA isomerase
FAR: Fatty acyl-CoA reductase
pgFAR: PG specific FAR
AR: Aldehyde reductase
FAT: Fatty acetyltransferase
AE: Antennal esterase
ORN: Olfactory receptor neuron
JTT model: Jones-Taylor-Thornton (JTT) model
UTR: Untranslated region
IS: Internal standard
