# Supplementary material for: Genes involved in sex pheromone biosynthesis of Ephestia cautella, an important food storage pest, are determined by transcriptome sequencing
Source: BMC Genomics. 2015 Jul 18;16(1):532. doi: 10.1186/s12864-015-1710-2 (PMC4506583; doi:10.1186/s12864-015-1710-2)
Supplement: Additional file 1: Figure S1. — Comparison of the E. cautella proposed pheromone biosynthetic pathway with those of Spodoptera exigua and S. littoralis. [file 12864_2015_1710_MOESM1_ESM.docx]

**Additional file 1: Figure S1**

Comparison of the *E. cautella* proposed pheromone biosynthesis pathway with those of *Spodoptera exigua* and *S. littoralis* (adopted from Munoz et al., 2008 and Acin et al., 2010). *S. exigua* and *S. littoralis* uses *Z*9,*E*12-14:OAc as one of the sex pheromone compound, and the major pheromone precursor is *Z*9-14:Acyl. We reported *E*12-14:Acyl as the major pheromone precursor in *E. cautella* and proposed the pheromone biosynthesis pathway (see Figure 3 legend for a detailed description).


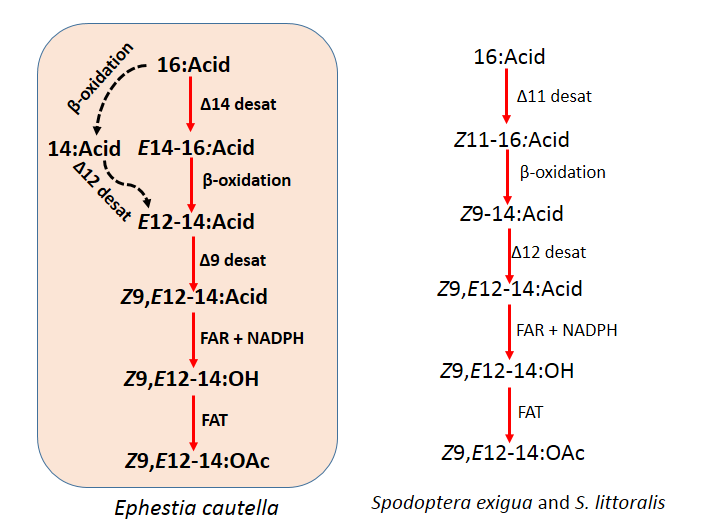


**References:**

Acín P, Rosell G, Guerrero A, Quero C. Sex pheromone of the Spanish population of the beet armyworm *Spodoptera exigua*. J Chem Ecol 2010;36(7):778-786.

Munoz L, Rosell G, Quero C, Guerrero A. Biosynthetic pathways of the pheromone of the Egyptian armyworm *Spodoptera littoralis*. Physiol Entomol. 2008;33(4):275-290.
