# Supplementary material for: Genes involved in sex pheromone biosynthesis of Ephestia cautella, an important food storage pest, are determined by transcriptome sequencing
Source: BMC Genomics. 2015 Jul 18;16(1):532. doi: 10.1186/s12864-015-1710-2 (PMC4506583; doi:10.1186/s12864-015-1710-2)
Supplement: Additional file 2: Table S2. — Comparative summary of E. cautella, A. ipsilon [39], Grapholita molesta [41], H.s virescens [38] and B. mori [38] PG transcriptome sequencing assemblies and annotations. [file 12864_2015_1710_MOESM2_ESM.docx]

**Additional file 2: Table S2.** Comparative summary of *E. cautella, A. ipsilon* [39]*, Grapholita molesta* [41], *H. virescens* [38] and *B. mori* [38] PG transcriptome sequencing assemblies and annotations.

|  | ***E. cautella**** | ***A. ipsilon**** | ***G. molesta**** | ***H. virescens***** | ***B. mori***** |
| --- | --- | --- | --- | --- | --- |
| **Raw reads** | 237,048,152 | 631,425 | 6,918,222,452 | NA | NA |
| **Clean reads** | 227,994,544 | 624,107 | NA | 17233 | 12296 |
| **Singletons/**  **Unassembled reads** | 65,928,240 | 169,721 | NA | 6228 | 2153 |
| **Number of contigs** | 83,792 | 17,508 | 104,463 | 2082 | 1794 |
| **Scaffolded metrics**  **-N50**  **-average**  **-Max**  **-Min** | 760 bp  590 bp  19,518 bp  107 bp | 1014 bp  892 bp  10,464 bp  77 bp | 1054 bp  553 bp  9815 bp  201 bp | NA  1100 bp  NA  NA | NA  NA  NA  NA |

**Next generation sequencing; ** Sanger sequencing; NA: not available*
