# Supplementary material for: Genes involved in sex pheromone biosynthesis of Ephestia cautella, an important food storage pest, are determined by transcriptome sequencing
Source: BMC Genomics. 2015 Jul 18;16(1):532. doi: 10.1186/s12864-015-1710-2 (PMC4506583; doi:10.1186/s12864-015-1710-2)

**Additional file 3: Figure S3**

**Characteristics of homology searches of *E. cautella* protein-coding genes against the non-redundant protein sequences (*nr*) at NCBI using Blastp.** **(A)** e-value distribution of the top BLAST hit for each unique sequence with a cut-off e-value of 1.0e-6. **(B)** Similarity distribution of the top BLAST hit for each unique sequence. The sequence similarity of *E. cautella* with database by Blast search ranges from 36% to approx. 100% and **(C)** Top-species distribution of the top BLAST hit for each unique sequence. The sequences of *E. cautella* sequences showed the most significant similarity to the sequences of *B. mori* followed by sequences of *D. plexippus*


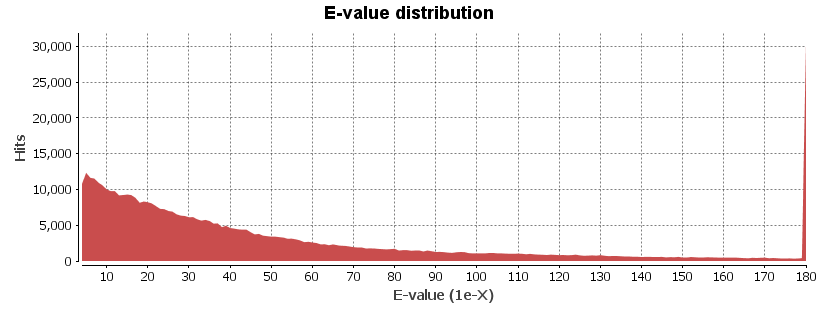


**A**


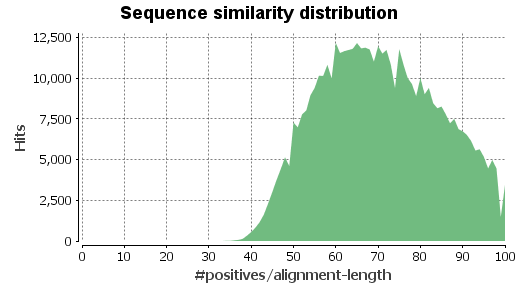


**B**

**C**


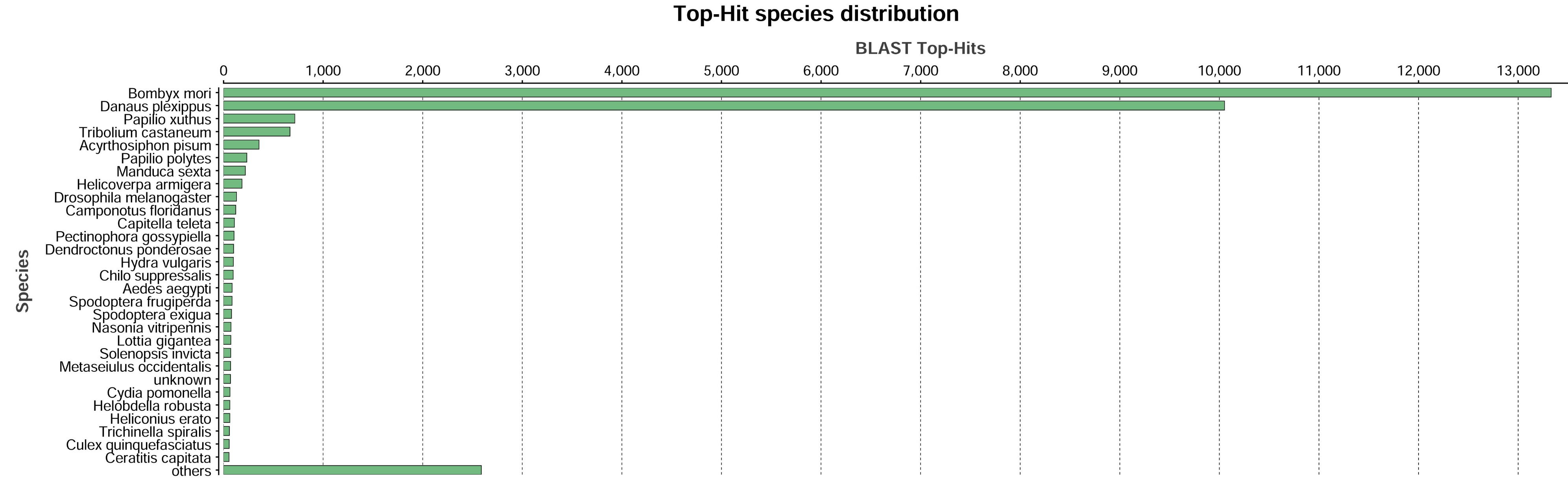

Supplement: Additional file 3: Figure S3. — Characteristics of homology searches of E. cautella protein-coding genes against the non-redundant protein sequences (nr) at NCBI using BLASTp. [file 12864_2015_1710_MOESM3_ESM.docx]
