# Supplementary material for: Genes involved in sex pheromone biosynthesis of Ephestia cautella, an important food storage pest, are determined by transcriptome sequencing
Source: BMC Genomics. 2015 Jul 18;16(1):532. doi: 10.1186/s12864-015-1710-2 (PMC4506583; doi:10.1186/s12864-015-1710-2)

**Additional file 4: Figure S4**

**Functional assignment terms to query sequences from the pool of GO terms gathered in the mapping step.** **(A)** Data distribution with 30,582 blast hits, 53,210 without blast hit, 4,217 with mapping results and 20,615 annotated sequences **(B)** Annotation distribution; Most sequences have between 1 and 6 GO terms annotated; **(C)** GO-level distribution. *E. cautella* sequence GO terms representation for biological process (BP), molecular function (MF) and cellular component (CC) ontologies. The mean GO-level is 4.385 and 97437 annotations could be assigned; **(D)** Number of GO-terms for *E. cautella* sequences with length (x). The length of most GO term annotated *E. cautella* sequences with average 590 bp; **(E)** Annotation score distribution; and **(F)** Percentage of *E. cautella* sequences with length (x) annotated.

**B**

**A**


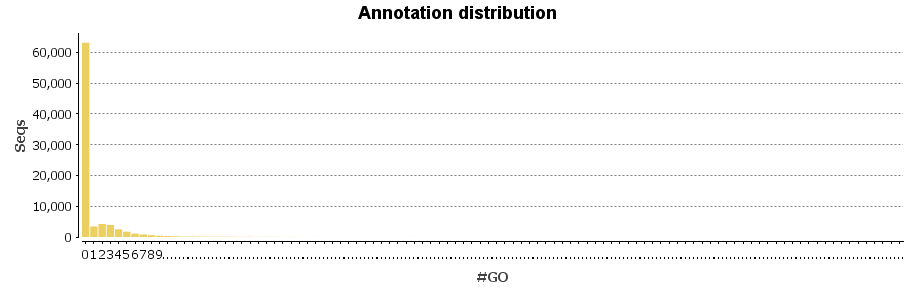


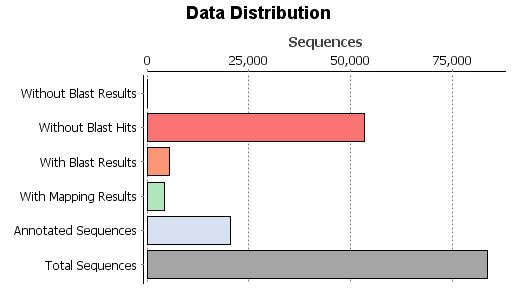


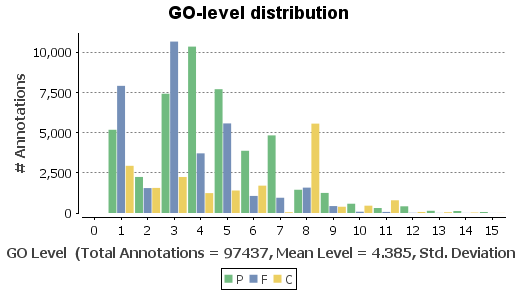


**D**.

**C**


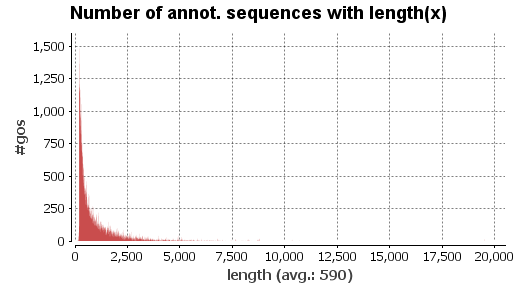


P: Biological process, F: Molecular function; C: cellular component

**F**

**E**


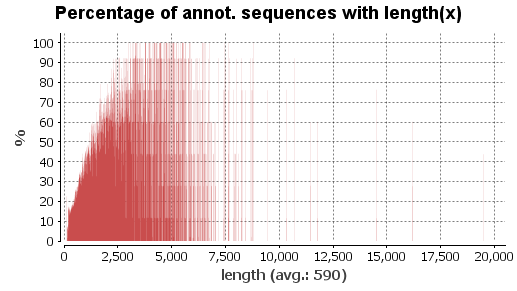


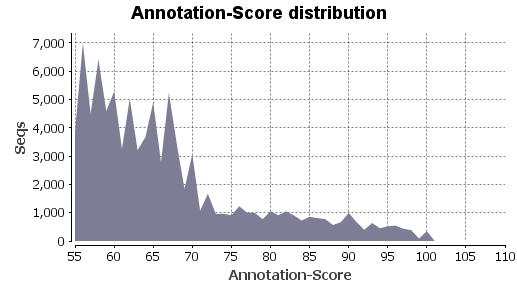

Supplement: Additional file 4: Figure S4. — Functional assignment terms to query sequences from the pool of GO terms gathered in the mapping step. [file 12864_2015_1710_MOESM4_ESM.docx]
