# Supplementary material for: Genes involved in sex pheromone biosynthesis of Ephestia cautella, an important food storage pest, are determined by transcriptome sequencing
Source: BMC Genomics. 2015 Jul 18;16(1):532. doi: 10.1186/s12864-015-1710-2 (PMC4506583; doi:10.1186/s12864-015-1710-2)

**Additional file 5: Figure S5**

**Mapping of *E. cautella* protein-coding genes to GO terms associated to BLASTp hits. (A) Evidence code distribution for BLAST hits.** The evidence code distribution for BLAST hits chart shows an overrepresentation of Inferred Electronic Annotation (IEA), followed by Inferred by Mutant Phenotype (IMP); **(B)** Evidence code distribution for individual sequences. The highest evidence code for the individual sequences was through Inferred Electronic Annotation (IEA), second by Inferred by Mutant Phenotype (IMP) and third by Inferred by Direct Assay (IDA); **(C)** Mapping database sources. The majority of *E. cautella* genes are obtained from the UniProt Knowledge Base (KB) followed by FlyBase (FB).


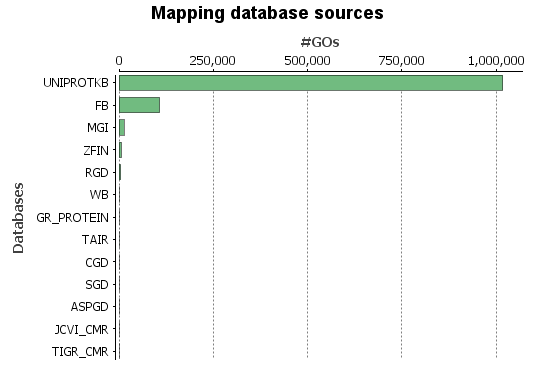


**B**

**C**

**A**


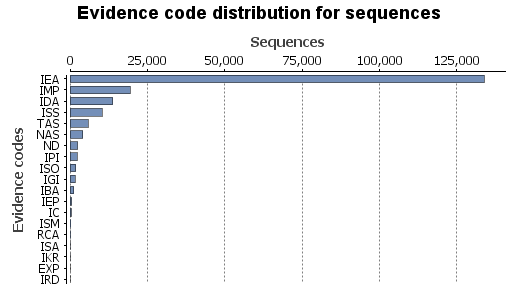


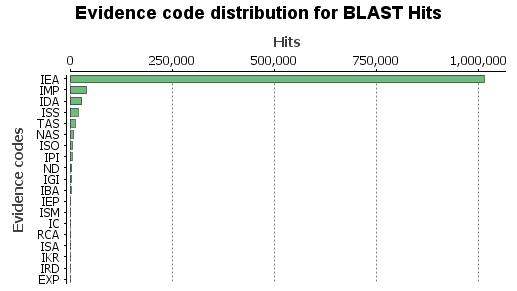

Supplement: Additional file 5: Figure S5. — Mapping of E. cautella protein-coding genes to GO terms associated to BLASTp hits. [file 12864_2015_1710_MOESM5_ESM.docx]
