# Supplementary material for: Genes involved in sex pheromone biosynthesis of Ephestia cautella, an important food storage pest, are determined by transcriptome sequencing
Source: BMC Genomics. 2015 Jul 18;16(1):532. doi: 10.1186/s12864-015-1710-2 (PMC4506583; doi:10.1186/s12864-015-1710-2)

**Additional file 6: Figure S6**

**KEGG pathway representing the biosynthesis of unsaturated fatty acids.** ∆9 desaturase, β-oxidation enzymes (Acyl-CoA oxidase, Acyl-CoA dehydrogenase, Enoyl-CoA hydratase) activities are shown in the pathway.


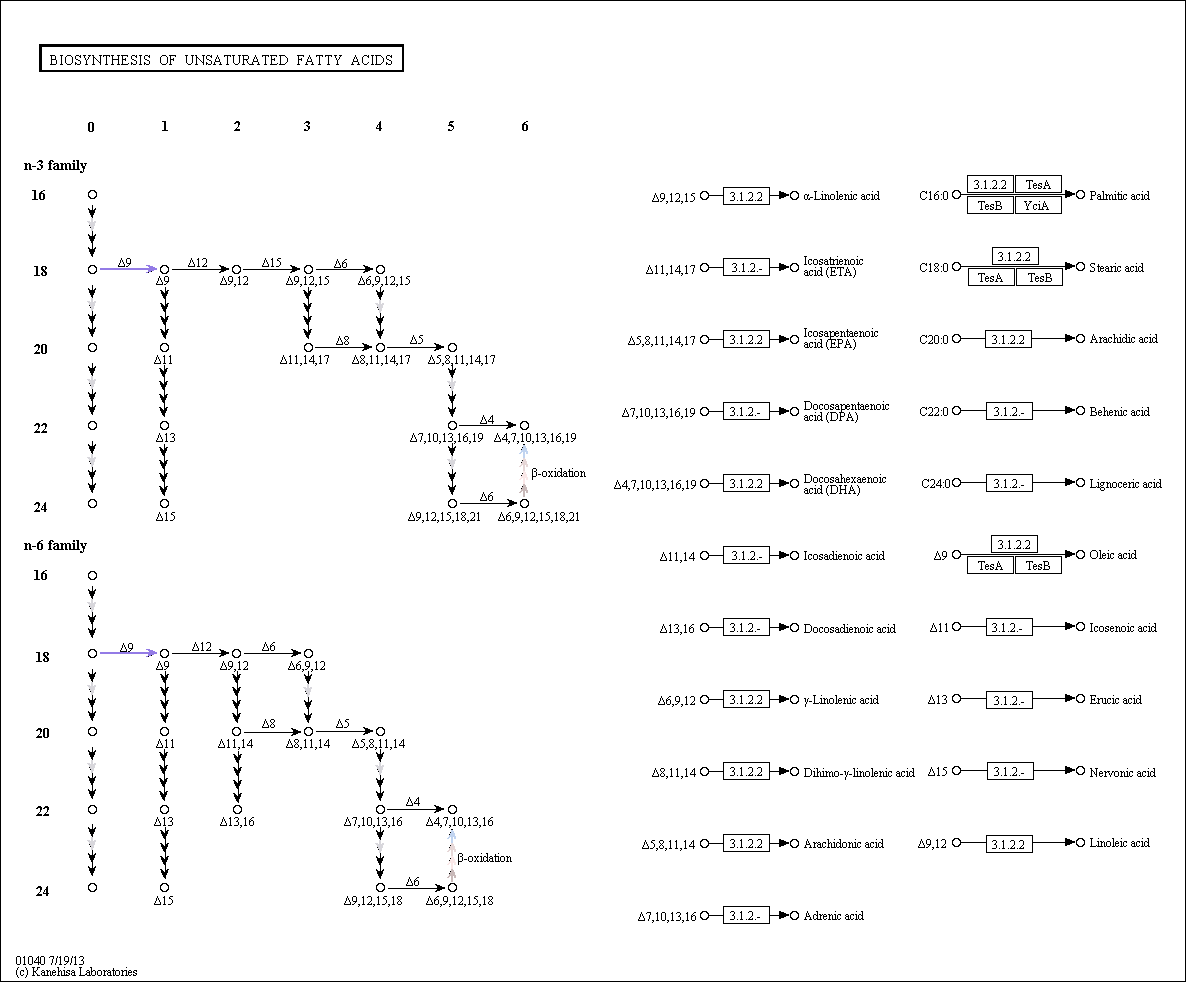

Supplement: Additional file 7: Figure S6. — KEGG pathway representing the biosynthesis of unsaturated fatty acids. ∆9 desaturase, β-oxidation enzymes (Acyl-CoA oxidase, Acyl-CoA dehydrogenase, Enoyl-CoA hydratase) activities are shown in the pathway. [file 12864_2015_1710_MOESM7_ESM.docx]
