# Supplementary material for: Genes involved in sex pheromone biosynthesis of Ephestia cautella, an important food storage pest, are determined by transcriptome sequencing
Source: BMC Genomics. 2015 Jul 18;16(1):532. doi: 10.1186/s12864-015-1710-2 (PMC4506583; doi:10.1186/s12864-015-1710-2)
Supplement: Additional file 8: Table S7. — Putative pheromone biosynthesis-related genes involved in β-oxidation in the E. cautella PG. [file 12864_2015_1710_MOESM8_ESM.pdf]

Additional file 7: Table S7 Putative pheromone biosynthesis-related genes involved in β-oxidation in the *E. cautella* PG

Unigene	Accession no.	Length (bp)	Putative identification	Species	Accession no.	Blast hit score	E-value	% of identity	RPKM
**Acyl Co-A dehydrogenase**									
EP_Unigene_1_ACD	GBXH01000033	415	fatty acid beta-oxidation complex subunit beta	*Danaus plexippus*	EHJ76018	274.633	1.40E-87	92.4	27
EP_Unigene_2_ACD	GBXH01000034	1938	acyl-coa dehydrogenase	*Papilio xuthus*	BAM17923	442.58	0	93.1	219
EP_Unigene_3_ACD	GBXH01000035	1601	hydroxyacyl-coenzyme A dehydrogenase	*Bombyx mori*	NP_001040132	802	0	82.2	271
EP_Unigene_4_ACD	GBXH01000036	1308	acyl-coenzyme A dehydrogenase	*Bombyx mori*	NP_001037672	648	0	79.8	180
EP_Unigene_5_ACD	GBXH01000037	513	acyl-dehydrogenase family member 9	*Agrotis segetum*	AID66671	207	8.43E-60	68.2	10
EP_Unigene_6_ACD	GBXH01000038	316	acyl-dehydrogenase family member 9	*Agrotis segetum*	XP_004926333	183	2.15E-52	73.8	7
EP_Unigene_7_ACD	GBXH01000039	981	short-chain specific acyl-mitochondrial	*Danaus plexippus*	EHJ71227	345	6.59E-113	93.9	25
EP_Unigene_8_ACD	GBXH01000040	780	mitochondrial aldehyde dehydrogenase	*Danaus plexippus*	EHJ66210	240	2.59E-109	83.3	198
EP_Unigene_9_ACD	GBXH01000041	643	Isovaleryl coenzyme a dehydrogenase	*Danaus plexippus*	EHJ70269	311	3.66E-101	91.8	29
EP_Unigene_10_ACD	GBXH01000042	453	acyl-dehydrogenase family member 9	*Agrotis segetum*	AID66671	187	6.28E-53	72.3	10
EP_Unigene_11_ACD	GBXH01000043	439	acyl-coa dehydrogenase	*Papilio xuthus*	BAM18207	276	8.37E-89	90.7	149
EP_Unigene_12_ACD	GBXH01000044	423	fatty acid beta-oxidation complex subunit	*Agrotis segetum*	AID66698	280	1.31E-89	93.4	41
EP_Contig_ 419_ACD	GBXH01000513	743	isovaleryl- mitochondrial	*Bombyx mori*	NP_001129356	377.481	1.23E-126	92	25
EP_Contig_ 2353_ACD	GBXH01002443	1888	glutaryl- mitochondrial-like	*Bombyx mori*	XP_004932115	760.755	0	94	38
EP_Contig_ 2541_ACD	GBXH01002631	1353	3-hydroxyacyl- dehydrogenase	*Agrotis segetum*	AID66694	423.32	6.86E-143	90	108
EP_Contig_ 2704_ACD	GBXH01002794	862	3-hydroxyacyl-coa dehyrogenase	*Danaus plexippus*	EHJ74477	271.552	4.04E-81	94	35
EP_Contig_ 7055_ACD	GBXH01007134	1506	very long-chain specific acyl- mitochondrial	*Danaus plexippus*	EHJ74455	827.395	0	91	3
EP_Contig_ 10512_ACD	GBXH01010577	451	3-hydroxyacyl- dehydrogenase	*Bombyx mori*	NP_001040414	127.872	1.19E-32	92	10
EP_Contig_ 11295_ACD	GBXH01011358	610	short-chain specific acyl- mitochondrial	*Danaus plexippus*	EHJ71227	231.106	2.72E-70	98	5
EP_Contig_ 12236_ACD	GBXH01012295	962	short-chain specific acyl- mitochondrial	*Bombyx mori*	XP_004932583	436.802	1.01E-148	91	25
EP_Contig_ 14211_ACD	GBXH01014264	1008	3-hydroxyacyl- dehydrogenase	*Bombyx mori*	NP_001040414	304.679	3.82E-98	89	87
EP_Contig_ 19220_ACD	GBXH01019261	572	3-hydroxyacyl- dehydrogenase	*Danaus plexippus*	EHJ68409	162.54	3.01E-45	91	43
EP_Contig_ 22027_ACD	GBXH01022052	256	fatty acid beta-oxidation complex subunit beta	*Agrotis segetum*	AID66698	170.244	1.96E-48	98	29
EP_Contig_ 22958_ACD	GBXH01022980	211	mitochondrial aldehyde dehydrogenase	*Bombyx mori*	NP_001040475	119.783	4.11E-30	88	15
EP_Contig_ 34107_ACD	GBXH01034076	234	acyl- dehydrogenase family member 9	*Agrotis segetum*	AID66671	128.642	7.11E-33	89	5
EP_Contig_ 35858_ACD	GBXH01035811	778	3-hydroxyacyl- dehydrogenase type-2-like	*Bombyx mori*	XP_004927100	181.03	2.16E-84	79	4
EP_Contig_ 36844_ACD	GBXH01036786	575	short-chain specific acyl- dehydrogenase	*Agrotis segetum*	AID66668	239.58	9.17E-74	92	18
EP_Contig_ 38509_ACD	GBXH01038442	1380	3-hydroxyacyl- dehydrogenase	*Agrotis segetum*	AID66692	425.631	1.61E-144	95	10
**Acyl Co-A oxidase**									
EP_Unigene_1_ACO	GBXH01000045	1785	acyl- oxidase	*Bombyx mori*	XP_004932402	806.979	0	81	192
EP_Unigene_2_ACO	GBXH01000046	665	probable peroxisomal acyl-coenzyme a oxidase 1-like	*Agrotis segetum*	AID66679	214.542	1.48E-73	83	78
EP_Unigene_3_ACO	GBXH01000047	3572	peroxisomal acyl-coenzyme a oxidase 3	*Agrotis segetum*	AID66676	1054.66	0	91	8
EP_Unigene_4_ACO	GBXH01000048	1561	probable peroxisomal acyl-coenzyme a oxidase 1-like	*Bombyx mori*	XP_004932404	365.54	3.54E-167	76	8
EP_Unigene_5_ACO	GBXH01000049	1349	acyl- oxidase	*Danaus plexippus*	EHJ63526	317.005	9.74E-163	76	40
EP_Unigene_6_ACO	GBXH01000050	1308	acyl-coa dehydrogenase	*Bombyx mori*	NP_001037672	648.277	0	89	127
EP_Unigene_7_ACO	GBXH01000051	1002	probable peroxisomal acyl-coenzyme a oxidase 1-like	*Danaus plexippus*	EHJ66979	352.443	1.10E-114	60	5
EP_Unigene_8_ACO	GBXH01000052	756	probable peroxisomal acyl-coenzyme a oxidase 1-like	*Bombyx mori*	XP_004932403	349.362	1.84E-112	79	19
EP_Unigene_9_ACO	GBXH01000053	703	probable peroxisomal acyl-coenzyme a oxidase 1-like	*Agrotis segetum*	AID66677	329.331	3.97E-108	85	25
EP_Unigene_10_ACO	GBXH01000054	643	isovaleryl coenzyme a dehydrogenase	*Danaus plexippus*	EHJ70269	310.842	3.66E-101	95	29
EP_Unigene_11_ACO	GBXH01000055	560	peroxisomal acyl-coenzyme a oxidase 3-like	*Agrotis segetum*	AID66678	310.457	3.22E-98	88	13
EP_Unigene_12_ACO	GBXH01000056	253	probable peroxisomal acyl-coenzyme a oxidase 1-like	*Bombyx mori*	XP_004932398	140.198	8.92E-37	88	4
EP_Contig_ 1593_ACO	GBXH01001684	203	peroxisomal acyl- oxidase 3	*Agrotis segetum*	AID66678	83.9593	6.21E-17	83	5
EP_Contig_ 6314_ACO	GBXH01006395	1366	probable peroxisomal acyl-coenzyme a oxidase 1	*Agrotis segetum*	AID66679	481.871	0	79	37
EP_Contig_ 12754_ACO	GBXH01012812	927	peroxisomal acyl-coenzyme a oxidase 3-like	*Agrotis segetum*	AID66678	397.127	1.10E-129	88	10
EP_Contig_ 43976_ACO	GBXH01043870	633	probable peroxisomal acyl-coenzyme a oxidase 1	*Danaus plexippus*	EHJ63526	80.1073	5.66E-14	77	10
EP_Contig_ 80594_ACO	GBXH01079713	293	probable peroxisomal acyl-coenzyme a oxidase 1-like	*Danaus plexippus*	EHJ63527	137.502	1.24E-35	80	0.1
**Enoyl-Co-A hydratase**									
EP_Unigene_1_ECH	GBXH01000087	415	fatty acid beta-oxidation complex subunit beta	*Danaus plexippus*	EHJ76018	274.633	1.40E-87	98	26

EP_Unigene_2_ECH	GBXH01000088	749	enoyl- hydratase	*Bombyx mori*	XP_004922585	375.17	7.53E-127	89	32
EP_Unigene_3_ECH	GBXH01000089	1393	3-hydroxyisobutyryl-CoA hydrolase isoform a	*Bombyx mori*	XP_004931208	615.15	0	93	63
EP_Unigene_4_ECH	GBXH01000090	1213	enoyl- hydratase	*Papilio xuthus*	BAM18079	539.65	0	93	323
EP_Unigene_5_ECH	GBXH01000091	1601	hydroxyacyl-coenzyme a dehydrogenase	*Bombyx mori*	NP_001040132	803.897	0	93	209
EP_Contig_2704_ECH	GBXH01002794	862	3-hydroxyacyl-coa dehyrogenase	*Danaus plexippus*	EHJ74477	271.552	4.04E-81	94	108
EP_Contig_4284_ECH	GBXH01004368	233	peroxisomal multifunctional enzyme type 2-like	*Danaus plexippus*	EHJ72407	83.1889	9.30E-17	97	59
EP_Contig_8714_ECH	GBXH01008788	796	4-hydroxybutyrate -transferase	*Danaus plexippus*	EHJ67185	273.478	5.38E-86	84	22
EP_Contig_9255_ECH	GBXH01009327	3482	n-sulphoglucosamine sulphohydrolase	*Bombyx mori*	XP_004922014	482.256	5.22E-154	90	17
EP_Contig_15289_ECH	GBXH01015340	868	cyclohex-1-ene-1-carboxyl- hydratase	*Agrotis segatum*	AID66690	410.223	7.69E-141	90	28
EP_Contig_22027_ECH	GBXH01022052	256	fatty acid beta-oxidation complex subunit beta	*Agrotis segatum*	AID66698	170.244	1.96E-48	98	32
EP_Contig_28042_ECH	GBXH01028044	214	trifunctional enzyme subunit mitochondrial-like	*Heliothis virescens*	ADB57045	144.05	6.41E-39	98	26
EP_Contig_28083_ECH	GBXH01028084	201	delta( )-delta( )-dienoyl- mitochondrial	*Bombyx mori*	XP_004923742	89.3521	8.31E-20	81	54
**L-3-hydroxyacyl-coenzyme A dehydrogenase**									
EP_Unigene_ 1_HCD	GBXH01082866	415	fatty acid beta-oxidation complex subunit beta	*Danaus plexippus*	EHJ76018	274.633	1.40E-87	98	25.5
EP_Unigene_ 2_HCD	GBXH01082867	1601	hydroxyacyl-coenzyme a dehydrogenase	*Bombyx mori*	NP_001040132	801.971	0	93	271
EP_Unigene_ 3_HCD	GBXH01082868	1883	peroxisomal multifunctional enzyme type 2 isoform x1	*Agrotis segatum*	AID66693	845.499	0	83	233
EP_Unigene_ 4_HCD	GBXH01082869	398	peroxisomal multifunctional enzyme type 2-like	*Danaus plexippus*	EHJ72407	97.0561	4.63E-21	96	542
EP_Contig_2541_HCD	GBXH01002631	1353	3-hydroxyacyl- dehydrogenase	*Agrotis segatum*	AID66694	423.32	6.86E-143	90	87
EP_Contig_2704_HCD	GBXH01002794	862	3-hydroxyacyl-coa dehyrogenase	*Danaus plexippus*	EHJ74477	271.552	4.04E-81	94	108
EP_Contig_5694_HCD	GBXH01005776	958	very-long-chain -3-hydroxyacyl-	*Bombyx mori*	XP_004929721	405.216	6.62E-137	80	259
EP_Contig_10512_HCD	GBXH01010577	451	3-hydroxyacyl- dehydrogenase	*Bombyx mori*	NP_001040414	127.872	1.19E-32	92	3
EP_Contig_14211_HCD	GBXH01014264	1008	3-hydroxyacyl- dehydrogenase	*Bombyx mori*	NP_001040414	304.679	3.82E-98	89	25
EP_Contig_16664_HCD	GBXH01016714	229	3-hydroxyacyl-coa dehyrogenase	*Danaus plexippus*	EHJ63930	72.7886	4.83E-14	94	234
EP_Contig_19220_HCD	GBXH01019261	572	3-hydroxyacyl- dehydrogenase	*Danaus plexippus*	EHJ68409	162.54	3.01E-45	91	43
EP_Contig_22027_HCD	GBXH01022052	256	fatty acid beta-oxidation complex subunit beta	*Agrotis segatum*	AID66698	170.244	1.96E-48	98	29
EP_Contig_23619_HCD	GBXH01023638	865	very-long-chain -3-hydroxyacyl-	*Drosophila erecta*	XP_001969616	227.639	4.37E-68	70	6
EP_Contig_29443_HCD	GBXH01029438	230	fatty acid beta-oxidation complex subunit beta	*Agrotis segatum*	AID66698	153.295	3.25E-42	97	10
EP_Contig_35858_HCD	GBXH01035811	778	3-hydroxyacyl- dehydrogenase type-2-like	*Bombyx mori*	XP_004927100	181.03	2.16E-84	79	4
EP_Contig_38509_HCD	GBXH01038442	1380	3-hydroxyacyl- dehydrogenase	*Agrotis segatum*	AID66692	425.631	1.61E-144	95	10
EP_Contig_66876_HCD	GBXH01066339	363	estradiol 17-beta-dehydrogenase	*Danaus plexippus*	EHJ72766	162.925	2.62E-48	91	0.2
**3-ketoacyl CoA-thiolase**									
EP_Unigene_2_KCA	GBXH01082871	415	fatty acid beta-oxidation complex subunit beta	*Danaus plexippus*	EHJ76018	274.633	1.40E-87	98	26
EP_Unigene_3_KCA	GBXH01082872	1351	3-ketoacyl- mitochondrial-like	*Bombyx mori*	XP_004926994	352.058	9.26E-114	74	1.8
EP_Unigene_4_KCA	GBXH01082873	423	fatty acid beta-oxidation complex subunit beta	*Agrotis segatum*	AID66698	280.026	1.31E-89	97	41
EP_Contig_2704_KCA	GBXH01002794	862	3-hydroxyacyl-coa dehyrogenase	*Danaus plexippus*	EHJ74477	271.552	4.04E-81	94	108
EP_Contig_7943_KCA	GBXH01008021	236	estradiol 17-beta-dehydrogenase 12	*Danaus plexippus*	EHJ79287	77.411	2.63E-15	73	6
EP_Contig_22027_KCA	GBXH01022052	256	fatty acid beta-oxidation complex subunit beta	*Agrotis segatum*	AID66698	170.244	1.96E-48	98	5
EP_Contig_27784_KCA	GBXH01027786	316	hydroxyacyl-coenzyme a dehydrogenase	*Bombyx mori*	NP_001040132	196.823	1.13E-56	96	79
EP_Contig_29774_KCA	GBXH01029769	270	sterol carrier protein 2 3-oxoacyl- thiolase	*Bombyx mori*	AAT39117	146.362	4.96E-40	87	3
**delta-3, delta-2 trans enoyl CoA Isomerase**									
EP_Unigene_ 1_TECI	GBXH01082834	638	enoyl-CoA delta isomerase 1	*Agrotis segatum*	AID66701	347.051	3.84E-117	87	177
EP_Contig_32154_TECI	GBXH01032136	709	peroxisomal -trans-enoyl- isomerase	*Bombyx mori*	XP_004931504	77	7.92E-70	77	25
EP_Contig_60736_TECI	GBXH01060354	785	enoyl-CoA delta isomerase 1	*Agrotis segatum*	AID66701	66	3.35E-69	66	0.72
**delta(3,5)-Delta(2,4)-dienoyl-CoA isomerase**									
EP1_contig_28083_DECI	GBXH01028084	201	delta(3,5)-Delta(2,4)-dienoyl-CoA isomerase,	*Bombyx mori*	XP_004923742	67	6e-20	64.06	26.1164
