# Supplementary material for: Genes involved in sex pheromone biosynthesis of Ephestia cautella, an important food storage pest, are determined by transcriptome sequencing
Source: BMC Genomics. 2015 Jul 18;16(1):532. doi: 10.1186/s12864-015-1710-2 (PMC4506583; doi:10.1186/s12864-015-1710-2)
Supplement: Additional file 9: Table S8 — Putative pheromone degrading enzymes in the E. cautella PG. [file 12864_2015_1710_MOESM9_ESM.pdf]

Additional file 8: Table S8 Putative pheromone degrading enzymes in the *E. cautella* PG

Unigene	Accession no.	Length (bp)	Putative identification	Species	Accession no.	Blast hit score	E-value	% of identity	RPKM
**Antennal Esterase**									
EP_Unigene_ 1_AE	GBXH01000057	2139	Antennal esterase cxe16	*Spodoptera littoralis*	ACV60243	696	0	64	39.70296
EP_Unigene_ 3_AE	GBXH01000058	1723	Esterase fe-4 like	*Spodoptera litura*	ABE01157	613	0	70.1	2.742484
EP_Unigene_ 4_AE	GBXH01000059	1633	Antennal esterase cxe11	*Spodoptera exigua*	AEJ38206	286	2.70E-83	56.2	2.662378
EP_Unigene_ 5_AE	GBXH01000060	1150	Antennal estarase cxe11	*Spodoptera exigua*	AEJ38206	422	2.61E-140	67.7	16.02636
EP_Unigene_ 6_AE	GBXH01000061	1042	Antennal esterase cxe5	*Bombyx mori*	NP_001121191	407	9.11E-135	73	4.528057
EP_Unigene_ 7_AE	GBXH01000062	865	Antennal esterase cxe18	*Spodoptera exigua*	AEJ38204	355	1.85E-115	85.4	33.07
EP_Unigene_ 8_AE	GBXH01000063	651	Antennal esterase cxe11	*Danaus plexippus*	EHJ68163	138	1.00E-34	55	10.65699
EP_Unigene_ 12_AE	GBXH01000064	1349	Carboxylesterase-6-like precursor	*Bombyx mori*	NP_001266383	402	3.00E-131	52	38.74642
EP_Unigene_ 14_AE	GBXH01000065	972	Antennal esterase cxe19	*Spodoptera littoralis*	ACV60246	436	1.24E-145	67.7	0.642417
EP_Unigene_ 15_AE	GBXH01000066	710	Esterase fe-4 like	*Bombyx mori*	XP_004925746	195	2.55E-54	52.8	0.371741
EP_Unigene_ 16_AE	GBXH01000067	673	Esterase fe-4 like	*Bombyx mori*	XP_004932947	159	9.00E-42	49	0.5
EP_Unigene_ 18_AE	GBXH01000068	286	Antennal esterase cxe3	*Chilo suppressalis*	ABD62772	85	2.63E-17	68.1	26.395
EP_Contig_ 15395_AE	GBXH01015446	268	Antennal esterase cxe13	*Spodoptera littoralis*	ACV60240	153	8.34E-42	79.5	36.97078
EP_Contig_ 15947_AE	GBXH01015998	222	Antennal esterase cxe10	*Spodoptera exigua*	AEJ38207	131	2.82E-34	86.8	10.09907
EP_Contig_ 17850_AE	GBXH01017898	425	Antennal esterase cxe13	*Spodoptera exigua*	ADR64701	199	9.95E-58	75.5	73.34342
EP_Contig_ 18657_AE	GBXH01018699	1796	Antennal esterase cxe18	*Spodoptera exigua*	AEJ38204	303	0	60.3	10.55373
EP_Contig_ 23278_AE	GBXH01023299	1532	Esterase fe-4 like	*Bombyx mori*	XP_004927161	524	8.55E-178	69.1	49.67051
EP_Contig_ 28382_AE	GBXH01028381	414	Antennal esterase cxe13	*Spodoptera littoralis*	ACV60240	108	1.60E-27	70.5	58.56542
EP_Contig_ 33761_AE	GBXH01033730	1712	Antennal esterase cxe16	*Spodoptera littoralis*	ACV60243	112	8.49E-23	74.1	5.020042
EP_Contig_ 38969_AE	GBXH01038897	311	Antennal esterase cxe4	*Spodoptera littoralis*	ACV60231	72	1.65E-12	71.8	18.39066
EP_Contig_ 39860_AE	GBXH01039784	1743	Carboxyl/cholinesterase-4A	*Bombyx mori*	BAI66480	158	2.00E-69	46	7.690047
EP_Contig_ 51966_AE	GBXH01051765	1286	Carboxyl/cholinesterase-3-precursor	*Bombyx mori*	NP_001165391	312	5.00E-97	60	1.715267
EP_Contig_ 56999_AE	GBXH01056703	239	antennal esterase CXE9	*Spodoptera littoralis*	ACV60236	74	9.10E-13	58	3.959071
EP_Contig_ 57310_AE	GBXH01057009	222	Antennal esterase cxe9	*Danaus plexippus*	EHJ68088	90	3.04E-19	69.2	5.348165
EP_Contig_ 57764_AE	GBXH01057450	910	Antennal esterase cxe18	*Spodoptera littoralis*	ACV60245	68	9.36E-10	54.2	0.410622
EP_Contig_ 57765_AE	GBXH01057451	711	Antennal esterase cxe5	*Spodoptera exigue*	ADR64702	275	6.03E-85	54.2	1.220631
EP_Contig_ 67903_AE	GBXH01067332	252	Esterase fe-4 like	*Bombyx mori*	XP_004924612	147	9.00E-40	81	0.286994
EP_Contig_ 81534_AE	GBXH01080625	342	Esterase fe-4 like	*Bombyx mori*	XP_004932947	110	7.99E-26	65.1	0.317204
EP_Contig_ 82246_AE	GBXH01081314	270	Antennal esterase cxe11	*Spodoptera exigue*	AEJ38206	124	2.10E-31	81.1	0.200896
EP_Contig_ 83328_AE	GBXH01082376	274	Antennal esterase cxe11	*Spodoptera exigue*	AEJ38206	58	9.72E-08	60.17	0.285946
EP_Contig_4899_AE	GBXH01004982	2334	carboxylesterase 3	*Bombyx mori*	NP_001040411	740.725	0	75	83.91392
EP_Contig_15948_AE	GBXH01015999	295	carboxyl choline esterase	*Spodoptera litura*	ABE01156	158.303	4.11E-47	84	24.16876
EP_Contig_16669_AE	GBXH01016719	215	odorant-degrading enzyme	*Spodoptera littoralis*	ABH01081	107.842	1.53E-25	78	61.72633
EP_Contig_21935_AE	GBXH01021961	859	carboxyl choline esterase	*Helicoverpa armigera*	ADF43499	63.5438	1.70E-09	71	0.406936
EP_Contig_33478_AE	GBXH01033450	1774	low quality protein: esterase b1-like	*Bombyx mori*	XP_004926539	742.265	0	80	1.579759
EP_Contig_35170_AE	GBXH01035128	1038	esterase fe4-like	*Bombyx mori*	XP_004933870	336.65	1.11E-107	81	11.0899
