# Supplementary material for: Genes involved in sex pheromone biosynthesis of Ephestia cautella, an important food storage pest, are determined by transcriptome sequencing
Source: BMC Genomics. 2015 Jul 18;16(1):532. doi: 10.1186/s12864-015-1710-2 (PMC4506583; doi:10.1186/s12864-015-1710-2)
Supplement: Additional file 10: Table S9. — Putative pheromone carrier proteins in the E. cautella PG. [file 12864_2015_1710_MOESM10_ESM.pdf]

Additional file 9: Table S9 Putative pheromone carrier proteins in the *E. cautella* PG

Unigene	Accession no.	Length (bp)	Putative identification	Species	Accession no.	Blast hit score	E-value	% of identity	RPKM
**Antennal binding proteins**									
EP_Unigene_ 1_ABP	GBXH01000027	529	Antennal binding protein 7	*Manduca sexata*	AAL60425	142	2.03E-39	72.6	57
EP_Unigene_ 2_ABP	GBXH01000028	330	Antennal binding protein 4	*Danaus plexippus*	EHJ65654	118	2.03E-31	72.7	0.3
EP_Contig_ 83004_ABP	GBXH01082060	275	Antennal binding protein	*Amyelois tranitella*	ACX47898	75	6.08E-15	92.4	0.17532703
**Odorant Binding proteins**									
EP_Unigene_ 1_OBP	GBXH01082874	492	Odorant binding protein 1	*Cnaphalocrocis medinalis*	AFG72998	219	1.22E-69	88.9	0.5
EP_Unigene_ 2_OBP	GBXH01082875	769	Odorant binding protein 17	*Helicoverpa armigera*	AFI57166	197	7.34E-60	91.5	29
EP_Unigene_ 3_OBP	GBXH01082876	522	Odorant binding protein 4	*Rhynchophorus palmarum*	AAQ96921	196	6.02E-61	73.9	0.5
EP_Unigene_ 4_OBP	GBXH01082877	502	Odorant binding protein 8	*Helicoverpa armigera*	AEB54589	129	8.49E-42	87.4	0.4
EP_Unigene_ 5_OBP	GBXH01082878	443	Odorant binding protein 2	*Batocera horsfieldi*	ADD70031	88	3.65E-19	64	0.4
EP_Unigene_ 6_OBP	GBXH01082879	428	General odorant binding protein 56d	*Tribolium castaneum*	XP_975684	136	1.01E-37	67	0.1
EP_Unigene_ 7_OBP	GBXH01082880	258	Odorant binding protein	*Dendroctonus ponderosae*	AFI45058	101	6.68E-25	63.4	0.1
EP_Unigene_ 8_OBP	GBXH01082881	253	Odorant binding protein 3	*Argyresthia conjugella*	AFD34180	132	9.44E-37	85.6	0.4
EP_Unigene_ 9_OBP	GBXH01082882	245	Odorant binding protein 1	*Chilo suppressalis*	AGM38605	129	4.94E-36	86.3	3
EP_Unigene_ 10_OBP	GBXH01082883	930	Generala odorant binding protein 1	*Amyelois transitella*	ACX47893	212	1.22E-64	90.9	3
EP_Unigene_ 11_OBP	GBXH01082884	560	Odorant binding protein 11	*Spodoptera exigua*	AGH70107	238	1.98E-76	59.5	2
EP_Contig_ 81136_OBP	GBXH01002388	291	odorant-binding protein 30	*Dendroctonus ponderosae*	AGI05176	108	9e-28	57.1	0.1
EP_Contig_ 2298_OBP	GBXH01006801	1804	Odorant binding protein	*Chilo suppressalis*	ADD71058	282	4.58E-87		70.4114424
EP_Contig_ 6721_OBP	GBXH01008535	1069	Odorant binding protein 16	*Helicoverpa armigera*	AFI57165	149	4e-40	61	103.076897
EP_Contig_ 8460_OBP	GBXH01047194	219	Odorant binding protein	*Chilo suppressalis*	ADD71058	82	4e-17	61.67	103.253236
EP_Contig_ 47339_OBP	GBXH01051917	484	Odorant binding protein 28	*Dendroctonus ponderosae*	AGI05178	71	8e-13	31.5	0.31720351
EP_Contig_ 52119_OBP	GBXH01080237	466	Odorant binding protein 13	*Dendroctonus ponderosae*	AGI05170	121	6e-32	48	1.12518756
**Chemosensory protein**									
EP_Unigene_ 1_CSP	GBXH01000076	777	Chemosensory protein 3	*Cnaphalocrocis medinalis*	AGI37365	101	5.16E-23	84.1	17.6
EP_Unigene_ 2_CSP	GBXH01000077	1425	chemosensory protein	*Chilo suppressalis*	AHC05672	111	1.10E-25	59.7	2.2
EP_Unigene_ 3_CSP	GBXH01000078	543	chemosensory protein	*Danaus plexippus*	EHJ70186	130	4.88E-35	80.5	168.2
EP_Unigene_ 4_CSP	GBXH01000079	608	chemosensory protein 3	*Agrotis ipsilon*	AGR39573		3e-39	62.3	14878
EP_Contig_ 3606_CSP	GBXH01000277	470	Chemosensory protein 4 variant	*Bombyx mori*	AFF18081	140	2e-39	60.6	6.6680227
EP_Contig_ 183_CSP	GBXH01003692	641	Chemosensory protein	*Danaus plexippus*	EHJ70186	127	2e-33	68.7	247.712682
EP_Contig_ 63373_CSP	GBXH01062946	664	Chemosensory protein	*Danaus plexippus*	EHJ67380	134	1e-35	76.9	0.43567711
**Pheromone Binding proteins**									
EP_Contig _27916_PBP	GBXH01027918	853	pheromone-binding protein 3-like	*Bombyx mori*	XP_00493386[illegible]	64	2e-09	54.33	19.1828171
EP_Contig_ 73451_PBP	GBXH01072743	422	pheromone-binding protein 1	*Amyelois transitella*	ACX47890	80	2e-26	89.7	0.14281675
