# Supplementary material for: Genes involved in sex pheromone biosynthesis of Ephestia cautella, an important food storage pest, are determined by transcriptome sequencing
Source: BMC Genomics. 2015 Jul 18;16(1):532. doi: 10.1186/s12864-015-1710-2 (PMC4506583; doi:10.1186/s12864-015-1710-2)
Supplement: Additional file 11: Table S10 — Putative receptor proteins in the E. cautella PG. [file 12864_2015_1710_MOESM11_ESM.pdf]

Additional file 10: Table S10 Putative receptor proteins in the *E. cautella* PG

Unigene	Accession no.	Length (bp)	Putative identification	Species	Accession no.	Blast hit score	E-value	% of identity	RPKM
**Odorant receptor**									
EP_Unigene_ 1_OR	GBXH01082894	444	Odorant partial	*Sesamia inferens*	AGY14589	60	3.05E-17	55	0.6
EP_Unigene_ 2_OR	GBXH01082895	1622	Odorant receptor 85c-like	*Bombyx mori*	SP_004925617	163	5.81E-07	51.9	1.5
EP_Unigene_ 3_OR	GBXH01082896	796	Odorant receptor 3 partial	*Planotortrix excessana*	ACN42950	307	5.95E-99	80.9	14
EP_Unigene_ 4_OR	GBXH01082897	533	Odorant receptor or12	*Cydia pomonella*	AFC91721	227	1.88E-69	72.4	0.4
EP_Unigene_ 5_OR	GBXH01082898	425	Odorant receptor 1	*Amyelois transitella*	AFP54146	127	4.66E-32	65.5	0.4
EP_Contig_ 440_OR	GBXH01000534	508	Odorant receptor partial OR43	*Cydia pomonella*	AFC91751	54	5.36E-07	77.22	34.35
EP_Contig_ 81292_OR	GBXH01005634	252	Odorant receptor partial 49	*Bombyx mori*	NP_001166614	60	3.31E-09	70.27	0.167412963
EP_Contig_ 5551_OR	GBXH01036898	585	Odorant receptor partial	*Cydia pomonella*	AFC91751	66	7.50E-11	59.62	34.77038466
EP_Contig_ 36956_OR	GBXH01069971	357	Odorant receptor partial	*Cydia pomonella*	AFC91751	55	2.79E-07	69.44	1.215502523
EP_Contig_ 70606_OR	GBXH01080391	284	Odorant receptor or12	*Cydia pomonella*	AFC91721	146	7.55E-40	65.96	0.169770892
EP_Unigene_ 1a_OR	GBXH01082885	475	Olfactory receptor 60	*Bombyx mori*	NP_001155301	208	5.11E-62	65.8	0.4
EP_Unigene_ 2a_OR	GBXH01082886	439	Olfactory receptor 12	*Helicoverpa armigera*	ACF32963	110	8.78E-26	77.6	0.3
EP_Unigene_ 3a_OR	GBXH01082887	1313	Olfactory receptor 50	*Manduca sexta*	AFL70813	307	3.59E-24	62.4	2
EP_Unigene_ 4a_OR	GBXH01082888	737	Olfactory receptor-like receptor	*Bombyx mori*	BAG12814	93	8.03E-19	66.4	2.011681414
EP_Unigene_ 5a_OR	GBXH01082889	366	Olfactory receptor 10	*Helicoverpa armigera*	ACC63238	154	4.81E-43	78.9	0.1
EP_Unigene_ 6a_OR	GBXH01082890	329	Olfactory receptor 19	*Spodoptera litura*	AGG08879	107	3.10E-25	64.3	0.2
EP_Unigene_ 7a_OR	GBXH01082891	315	Olfactory receptor 19	*Spodoptera litura*	AGG08879	82	3.31E-16	73.9	0.3
EP_Unigene_ 8a_OR	GBXH01082892	277	Olfactory receptor 12	*Danaus plexippus*	EHJ65925	76	1.36E-14	63.5	0.1
EP_Unigene_ 9a_OR	GBXH01082893	267	Olfactory receptor 10	*Helicoverpa armigera*	ACC63238	76	1.82E-14	67.3	0.2
EP_Contig_ 29799_OR	GBXH01029793	1627	Olfactory receptor 29	*Danaus plexippus*	EHJ78030	378	4.91E-118	68.77	9.127350637
EP_Contig_ 40311_OR	GBXH01040230	1244	Olfactor receptor	*Helicoverpa armigera*	AGK90007	158	3.10E-40	28.1	2.30610011
**Sensory neuron membrane protein**									
EP_Contig_ 1371_SNMP	GBXH01001464	1880	Sensory neuron membrane protein 2	*Ostrinia nubilalis*	ADQ73889	230	0	70	120.3674271
EP_Contig_ 25158_SNMP	GBXH01025173	2136	sensory neuron membrane protein 1	*Ostrinia nubilalis*	ADQ73892	780	0	73.71	1.221738422
**Ionotropic receptor**									
EP_Contig_ 46790_IR	GBXH01027302	1886	putative ionotropic receptor IR25a	*Cydia pomonella*	AFC91757	186	0	90.72	1.345339804
EP_Contig_ 27297_IR	GBXH01046655	566	ionotropic kainate 2 precursor	*Danaus plexippus*	EHJ66775	149	5.00E-40	78.4	4.557418616
EP_Contig_ 71353_IR	GBXH01070699	319	Ionotropic receptor partial	*Cydia pomonella*	AFC91759	213	1.09E-63	98.6	0.340073982
