# Supplementary material for: Genes involved in sex pheromone biosynthesis of Ephestia cautella, an important food storage pest, are determined by transcriptome sequencing
Source: BMC Genomics. 2015 Jul 18;16(1):532. doi: 10.1186/s12864-015-1710-2 (PMC4506583; doi:10.1186/s12864-015-1710-2)
Supplement: Additional file 12: Figure S11. — Maximum likelihood (ML) tree of the chemosensory proteins (CSPs). [file 12864_2015_1710_MOESM12_ESM.docx]

**Additional file 11: Figure S11**

**Maximum likelihood (ML) tree of the chemosensory proteins (CSPs).** *Bomby mori* CSPs [89] were used as reference to identify the *E. cautella* CSPs and the ML analysis was computed using MEGA (v.6.0) [103] (JTT model for ML heuristic searches methods was nearest-neighbor-interchange). *A. ipsilon* CSPs [39] and *H. virescens* CSPs [38] were also used to compare with *E. cautella* CSPs. *E. cautella* CSP transcripts are marked with blue bubble. *B. mori* CSP5 [89], *H. virescens* CSP (ACX53762) [38] and *A. ipsilon* CSP8 [39] are shown in red bubble. GenBank accession numbers are indicated.
