# Supplementary material for: Genes involved in sex pheromone biosynthesis of Ephestia cautella, an important food storage pest, are determined by transcriptome sequencing
Source: BMC Genomics. 2015 Jul 18;16(1):532. doi: 10.1186/s12864-015-1710-2 (PMC4506583; doi:10.1186/s12864-015-1710-2)

**Additional file 12: Figure S12**

**Maximum likelihood (ML) tree of the OR proteins.** *Helicoverpa armigera* ORs [92] and *Heliothis virescens* HR2, HR6 and H13 [77] were used as reference to identify the *E. cautella* ORs and the ML analysis was computed using MEGA (v.6.0) [103] (JTT model for ML heuristic searches methods was nearest-neighbor-interchange). The branch containing *H. armigera* OR83b was used as outgroup to root the tree. *E. cautella* transcripts are marked with blue bubble. *H. virescens* H13 and *H. armigera* OR13 are shown in red bubble. GenBank accession numbers are indicated. Scale 3.0 amino acid substitutions per site.


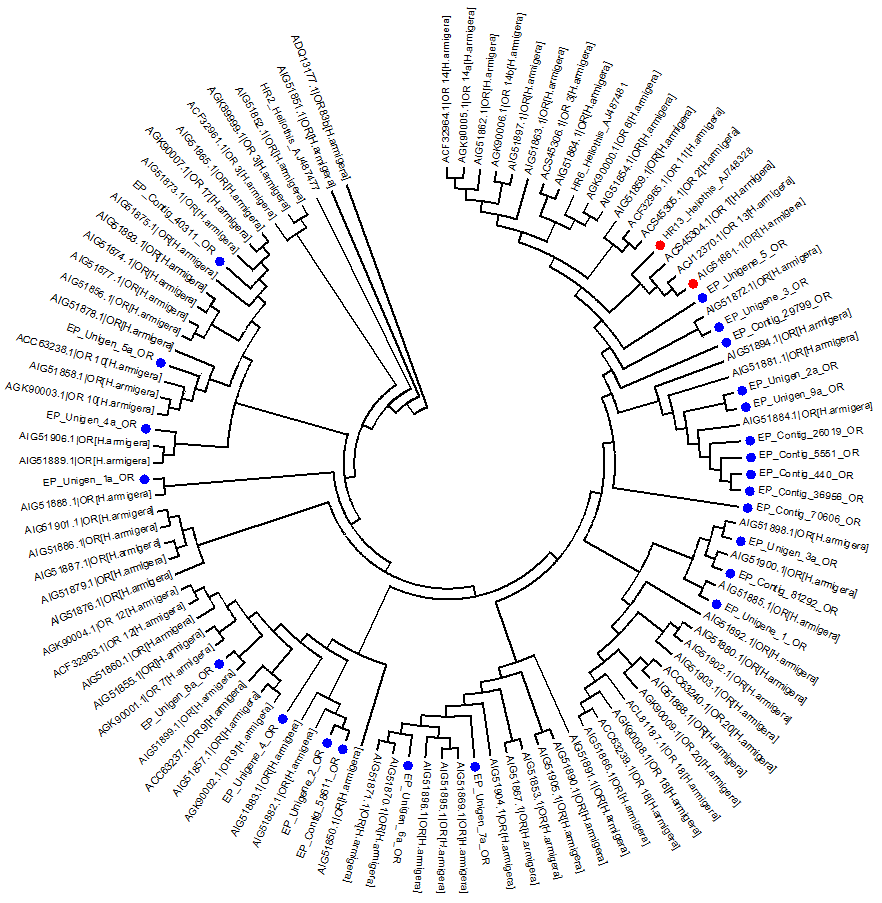

Supplement: Additional file 13: Figure S12. — Maximum likelihood (ML) tree of the OR proteins. [file 12864_2015_1710_MOESM13_ESM.docx]
